# Tuning the solid-state emission of liquid crystalline nitro-cyanostilbene by halogen bonding

**DOI:** 10.3762/bjoc.17.13

**Published:** 2021-01-14

**Authors:** Subrata Nath, Alexander Kappelt, Matthias Spengler, Bibhisan Roy, Jens Voskuhl, Michael Giese

**Affiliations:** 1 Organic Chemistry, University of Duisburg Essen, Universitätsstraße 7, 45141 Essen, Germany

**Keywords:** fluorescence, halogen bonding, liquid crystal

## Abstract

The first example of halogen-bonded fluorescent liquid crystals based on the interaction of iodofluorobenzene derivatives with nitro-cyanostilbenes is reported. The systematic variation of the fluorination degree and pattern indicates the relevance of the halogen bond strength for the induction of liquid crystalline properties. The modular self-assembly approach enables the efficient tuning of the fluorescence behaviour and mesomorphic properties of the assemblies.

## Introduction

Supramolecular chemistry has proven to be an efficient approach for the development of novel smart materials, since it relies on non-covalent interactions, which allow for dynamic responses to external stimuli [1]. In addition, the self-assembly of the complementary molecular entities provides an easy access to functional systems and enables recyclability and self-healing properties of the materials [2]. With respect to the formation of supramolecular liquid crystals, especially hydrogen bonding and halogen bonding have gained considerable attention [3–7]. In 2004, Bruce and co-workers reported the first example of a halogen-bonded liquid crystal based on pentafluoroiodobenzene and 4-alkoxystilbazole [5]. Ever since, several other groups employed halogen bonding for the formation of liquid crystalline materials [8–9]. For instance, Palacio et al. used (*E*)-1-(4-(octyloxy)phenyl)-2-(2,3,5,6-tetrafluoro-4-iodophenyl)diazene as a photo-switchable halogen bond donor and investigated the light-induced phase transition of the complexes with 4-alkoxystilbazoles [10]. Recently, Li and co-workers reported on a series of halogen-bonded assemblies to induce chirality in nematic liquid crystalline hosts and studied the light-induced manipulation of the photonic properties of these materials [11] In 2019, our group investigated the role of fluorine substitution of the aromatic halogen bond donor on the liquid crystallinity and the photo-response of halogen-bonded liquid crystals [12]. However, all reported halogen-bonded liquid crystals rely on the halogen-bond-acceptor capability of pyridyl units and so far, no study on the fluorescence behaviour of halogen-bonded liquid crystals has been reported.

**Figure 1:**
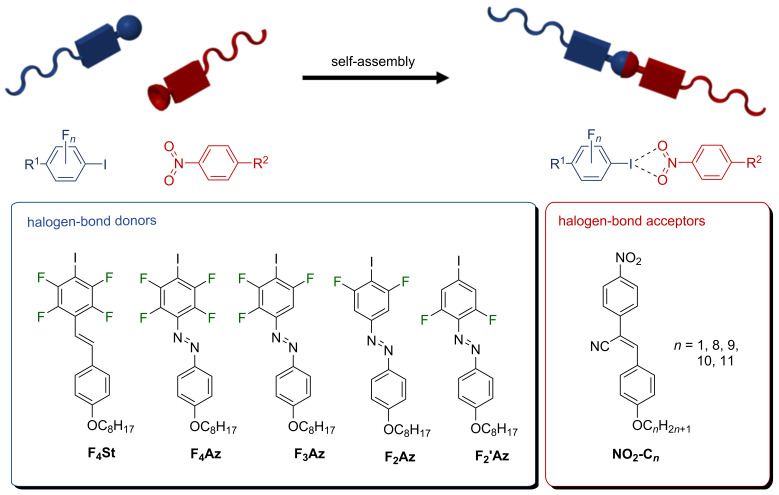
Schematic representation of the modular approach towards halogen-bonded fluorescent liquid crystals.

In 2014, Tothadi and Desiraju reported on a new supramolecular synthon based on the non-covalent interaction between 1,4-dinitrobenzene and iodobenzene [13]. Their structural analysis of a series of ternary cocrystals revealed that the nitro group is a suitable halogen bond acceptor, which interacts with polarised iodobenzene components in three different geometries – symmetrical, unsymmetrical or sidewise. Many other crystal structures support these findings and suggest the suitability of this synthon for the construction of supramolecular entities [14–17]. However, no examples for supramolecular materials employing this complementary interaction have been reported so far. In the present study we report the first halogen-bonded liquid crystal based on the complementary binding of nitro-cyanostilbene and tetrafluoroiodobenzene derivatives. Therefore, tetrafluoroiodostilbene (**F_4_St**) and a series of fluoroiodoazobenzenes (**F_4_Az**, **F_3_Az**, **F_2_Az**, **F_2_^’^Az**) were employed as halogen bond donors and combined with nitro-cyanostilbene (**NO_2_-C*_n_***) as fluorescent halogen bond acceptor (see Figure 1) to form halogen-bonded liquid crystals. The series of fluoroiodoazobenzenes with varying fluorination degree at the iodobenzene moiety was used to investigate the impact of halogen bonding on the properties of the assemblies. Since cyanostilbene molecules are known to show aggregation-induced emission (AIE) behaviour the photophysical properties of the resulting assemblies were investigated via variable-temperature fluorescence spectroscopy [18].

## Results and Discussion

The halogen-bonded assemblies were obtained by mixing the acceptor components **NO_2_-C*_n_*** with the donor entities **F_4_St** or **F_4_Az** in a 1:1 molar ratio in CH_2_Cl_2_. The slow evaporation of the solvent and subsequent drying in vacuo yielded the desired assemblies.

**Figure 2:**
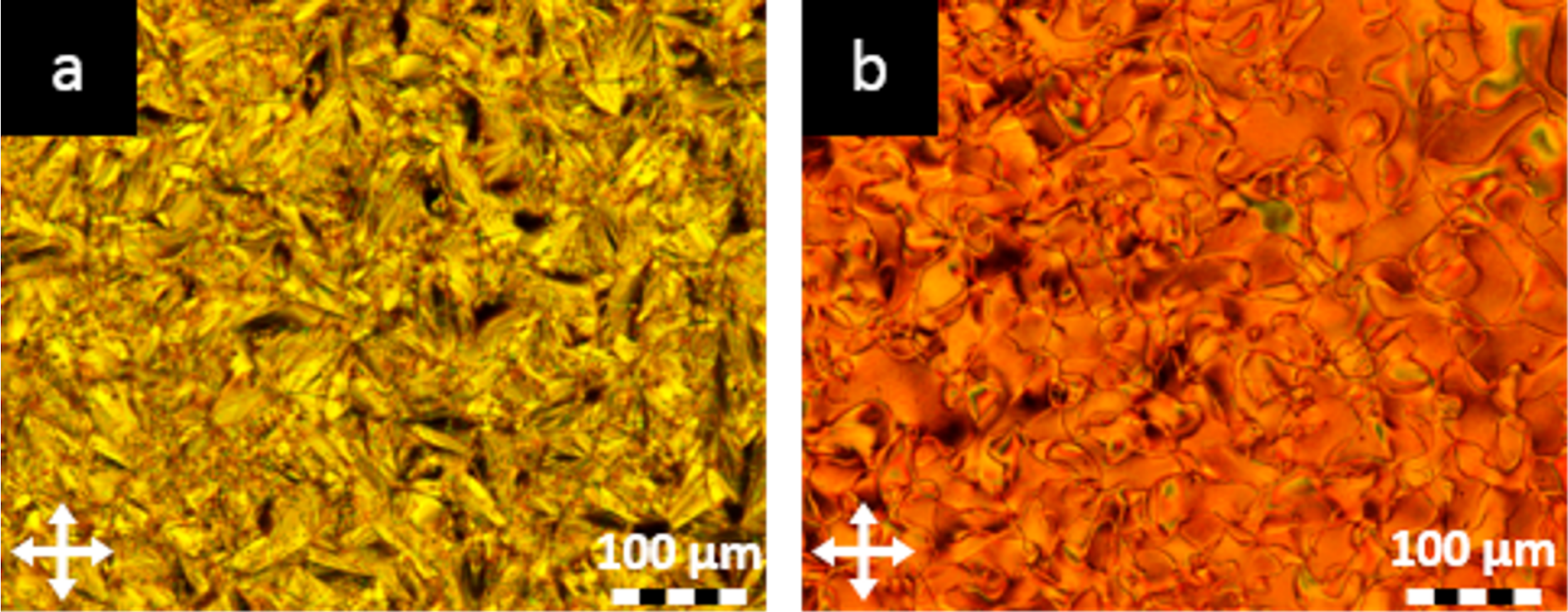
Representative POM images of **NO_2_-C_10_** at 94 °C (a) and **NO_2_-C_10_**∙∙∙**F_4_Az** at 61.5 °C (b) upon cooling from the isotropic melt.

The mesomorphic behaviour of the assemblies was investigated by polarised optical microscopy (POM) and differential scanning calorimetry (DSC). It should be noted that the individual building blocks **F_4_St** and **F_4_Az** as well as **NO_2_-C_8_** and **NO_2_-C_9_** do not exhibit mesomorphic behaviour. In contrast, mesophases were observed for components employing **NO_2_-C*_n_*** with longer alkyl chains. **NO_2_-C_10_** showed focal-conic textures at 94 °C during cooling (see Figure 2a) and **NO_2_-C_11_** showed an enantiotropic smectic behaviour (see Supporting Information File 1, Figures S11 and S15*).*

**Figure 3:**
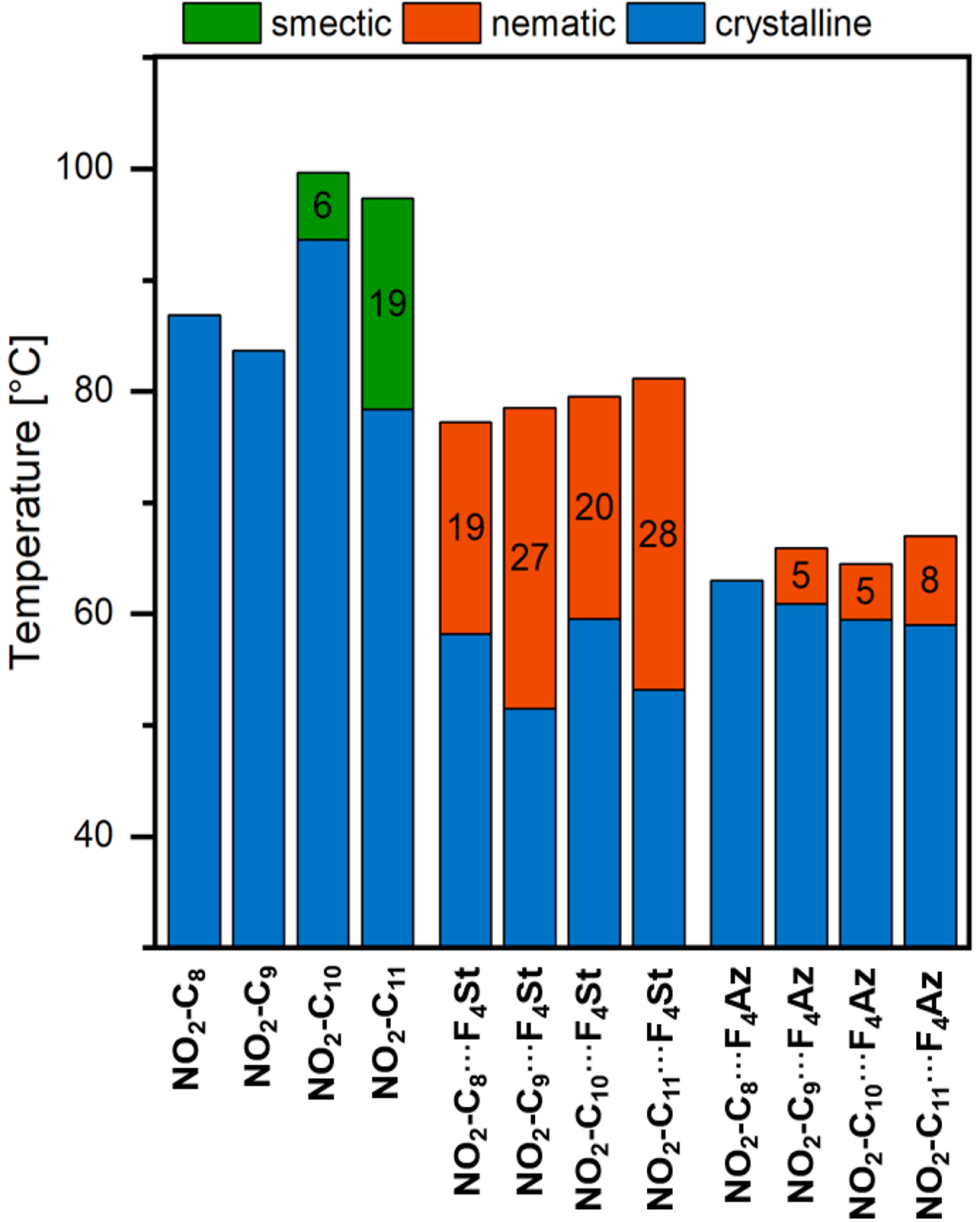
Comparison of the mesomorphic properties of **NO_2_-C*_n_***, **NO_2_-C*_n_***∙∙∙**F_4_St**, and **NO_2_-C*_n_***∙∙∙**F_4_Az** (*n* = 8–11). The transition temperatures were obtained by DSC upon cooling from the isotropic state. Mesophase ranges are given in black numbers.

In our initial set of assemblies, we combined **F_4_St** with **NO_2_-C*_n_*** with varying alkoxy-chain lengths (*n* = 8, 9, 10 and 11). The halogen-bonded assemblies exhibited mesogenic behaviour starting with an alkoxy chain length of *n* = 8. POM investigations revealed nematic mesophases for all complexes (see also Figure 2b) which is in contrast to the behaviour of pristine **NO_2_-C*_n_*** showing a smectic phase for the alkoxy chain lengths of *n* = 10 or 11. A strong odd–even effect was observed for the **NO_2_-C*_n_***∙∙∙**F_4_St** assemblies which indicates a significant impact of the alkyl chain length on the packing of the supramolecular entities in the solid state. This effect was also confirmed by the fluorescence behaviour (see paragraph on photophysical properties) and affects mainly the transition from the nematic to crystalline phase. In addition, it was observed that only assemblies with an odd number of carbon atoms in the alkoxy chain on the halogen bond accepting moiety (**NO_2_-C_9_** and **NO_2_-C_11_**) displayed an enantiotropic phase behaviour. For the **NO_2_-C*_n_***∙∙∙**F_4_Az** assemblies no significant effect of the alkoxy chain length on the transition temperatures was observed. The temperature ranges of the nematic phases are significantly narrower and liquid crystallinity is induced starting with **NO_2_-C_9_**∙∙∙**F_4_Az** (see Figure 3). The reduced performance of the azo series compared to the stilbazole series is in line with previous reports and can be attributed to the repulsion of the free electron pairs of the azo group [7]. Interestingly, enantiotropic phase transitions were observed exclusively for **NO_2_-C_10_**∙∙∙**F_4_Az**, indicating a different solid-state arrangement of the azo compounds compared to the stilbazole-based assemblies. The mesomorphic properties of all nitro compounds and the assemblies are summarised in Figure 3 and Table 1.

**Table 1:** Transition enthalpies of all compounds and assemblies upon heating and cooling as determined by DSC (heating/cooling rate of 10 °C/min).

component	heating				cooling		
		
	transition^a^	*T* (°C)	Δ*H* (kJ/mol)		transition^a^	*T* (°C)	Δ*H* (kJ/mol)

**NO_2_-C_8_**	Cr_1_→Cr_2_ Cr_2_→I	56.1 92.8	−13.6 43.4		I→Cr	85.9	−22.9
**NO_2_-C_9_**	Cr→I	86.9	22.7		I→Cr_1_ Cr_1_→Cr_2_	83.7 74.9	−20.6 −0.5
**NO_2_-C_10_**	Cr→I	100.9	61.6		I→Sm Sm→Cr_1_ Cr_1_→Cr_2_	99.4 93.6 84.6	−2.3 −57.7 −0.1
**NO_2_-C_11_**	Cr_1_→Cr_2_ Cr_2_→N N→I	59.9 81.2 96.3	0.4 20.9 1.8		I→Sm Sm→Cr	97.5 78.4	21.0 1.8
**NO_2_-C_8_**∙∙∙**F_4_St**	Cr_1_→Cr_2_ Cr_2_→Cr_3_ Cr_3_ → I	71.1 74.4 78.9	25.3 −2.4 35.4		I→N N→Cr	77.0 58.2	−1.0 −52.2
**NO_2_-C_9_**∙∙∙**F_4_St**	Cr→N N→I	65.1 77.3	46.8 0.8		I→N N→Cr	78.3 51.5	−0.9 −44.5
**NO_2_-C_10_**∙∙∙**F_4_St**	Cr_1_→Cr_2_ Cr_2_ → Cr_3_ Cr_3_→I	47.4 71.6 76.0	−3.3 43.0 41.6		I→N N→Cr	79.3 59.5	−1.3 −66.2
**NO_2_-C_11_**∙∙∙**F_4_St**	Cr_1_→Cr_2_ Cr_2_→Cr_3_ Cr_3_→N N→I	62.1 63.0 72.7 80.3	57.7 −7.8 14.6 0.9		I→N N→Cr	81.4 53.2	−1.3 −51.0
**NO_2_-C_8_**∙∙∙**F_4_Az**	Cr_1_→Cr_2_ Cr_2_→I	67.3 74.7	12.0 34.0		I→Cr	63.0	−45.1
**NO_2_-C_9_**∙∙∙**F_4_Az**	Cr→I	75.4	49.6		I→N N→Cr	65.8 60.9	−2.5 −41.6
**NO_2_-C_10_**∙∙∙**F_4_Az**	Cr_1_→Cr_2_ Cr_2_→N N→I	66.3 76.4 83.4	3.5 51.3 0.4		I→N N→Cr	64.1 59.5	−2.3 −47.5
**NO_2_-C_11_**∙∙∙**F_4_Az**	Cr_1_→Cr_2_ Cr_2_→I	66.4 77.9	2.0 51.7		I→N N→Cr_1_ Cr_1_→Cr_2_	67.1 59.0 50.9	−1.2 −41.5 −1.7
**NO_2_-C_10_**∙∙∙**F_3_Az**	Cr_1_→Cr_2_ Cr_2_→I	47.9 75.5	−21.1 85.4		I→N N→Cr	61.0 48.4	−0.8 −51.6
**NO_2_-C_10_**∙∙∙**F_2_Az**	Cr_1_→Cr_2_ Cr_2_→I	46.0 60.4	−10.4 82.0		I→Cr	54.4	−64.7
**NO_2_-C_10_**∙∙∙**F_2_’Az**	Cr_1_→Cr_2_ Cr_2_→I	46.1 59.6	−17.2 63.7		I→Cr	43.6	−42.8

^a^Cr: crystal, N: nematic, Sm: smectic, I: isotropic.

The strength of the halogen bond has a crucial impact on the formation of the liquid crystalline phase as shown, e.g., by Bruce et al. [5,19]. They reported on the thermal properties of halogen-bonded assemblies between stilbazoles and iodo- or bromopentafluorobenzene. While the iodo derivative formed assemblies with liquid crystalline properties, no LC behaviour was observed for the assemblies with bromopentafluorobenzene as it forms a weaker halogen bond. Related systems have been investigated by Yu and co-workers, who studied a series of halogen-bonded liquid crystals based on the combination of azopyridines with molecular iodine or bromine [9]. Interestingly, the broadest mesophase temperature ranges were found for the bromine-based assemblies and not as anticipated, for the iodine system which yields a stronger halogen bond.

**Figure 4:**
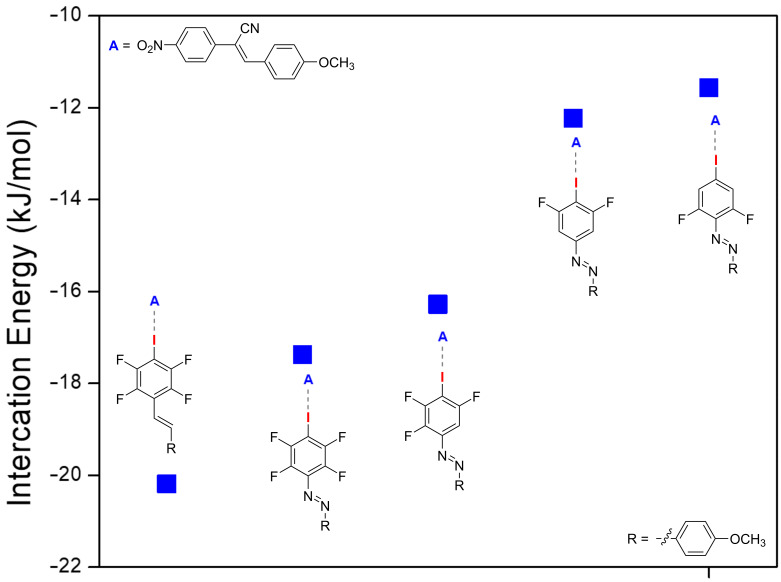
Graphical representation of the calculated interaction energies in kJ/mol of the XB-acceptor **NO_2_-C_1_** with **F_4_St**, **F_4_Az**, **F_3_Az**, **F_2_Az**, and **F_2_’Az**. For computational efficiency, the octyl chains have been replaced by methoxy groups.

In order to prove that the halogen bond plays a crucial role for the induction of liquid crystallinity in our assemblies, we synthesised a series of azo compounds with decreasing fluorination degree at the halogen bond donating iodobenzene [12]. Reducing the number of the fluorine atoms at the halogen bond donating moiety lowers the polarisation of the iodine atom and thus weakens the halogen bond. In a first step, we calculated the interaction energies of the assemblies of the azobenzene halogen bond donors **F_4_St**, **F_4_Az**, **F_3_Az**, **F_2_Az** or **F_2_’Az** with **NO_2_-C_1_**. For computational efficiency reasons the terminal alkoxy chains were substituted by methoxy groups. The interaction energies were calculated using the latest theoretical counterpoise correction on the mp2/LanL2DZ with the basis set super position error (BSSE). The computed interaction energies for the assemblies (Figure 4, Table S1 in Supporting Information File 1) decrease in the order **F_4_St** > **F_4_Az** > **F_3_Az** > **F_2_Az** > **F_2_’Az**. This is in accordance with our previous results on pyridine-based assemblies and supports the assumption that a reduction of the fluorination degree at the iodobenzene yields a weaker polarisation of the iodine and thus a weaker halogen bond [12].

To investigate this effect experimentally, a series of azo benzenes with different fluorination patterns was synthesised (**F_4_Az**, **F_3_Az**, **F_2_Az**, **F_2_’Az**) and combined with **NO_2_-C_10_** in CH_2_Cl_2_. Upon removal of the solvent the material was investigated with respect to its mesomorphic behaviour using POM and DSC. In a simplified view, the experimental data is in line with the theoretical data and confirms that the formation of thermally stable halogen bonding induces the liquid crystalline properties of the assemblies. We suppose that the formation of the halogen-bonded assembly expands the mesogenic core and yields a more balanced ratio of rigid and flexible segments, which is crucial for the formation of a mesophase [7,20].

**Figure 5:**
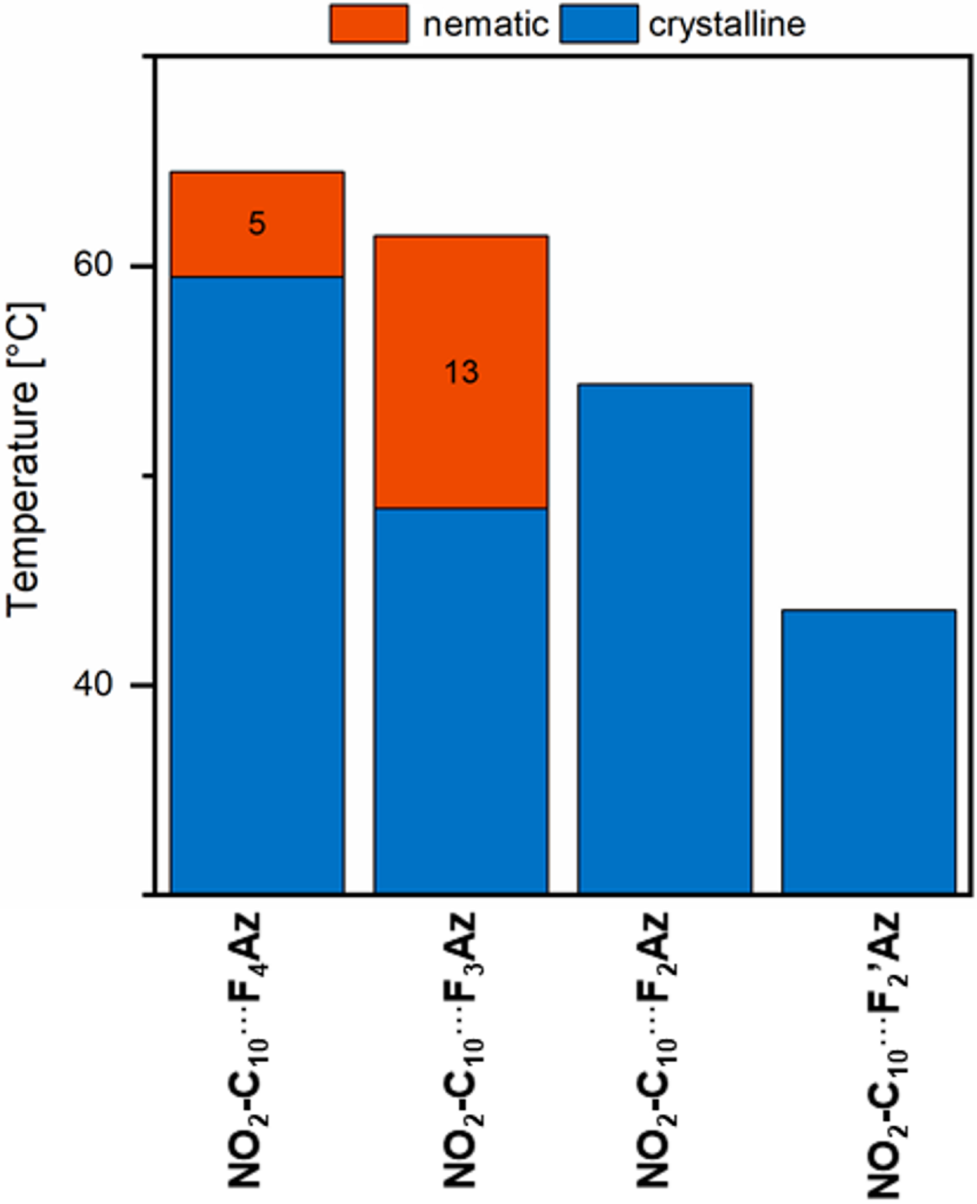
Summary of the thermal behaviour of the azo complexes with decreasing fluorination degree as observed by DSC upon cooling from the isotropic melt. Mesophase ranges are given in black numbers.

**Figure 6:**
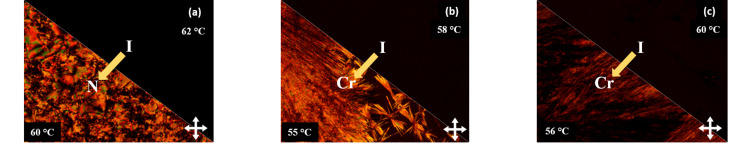
POM images of the supramolecular assemblies **NO_2_-C_10_**∙∙∙**F_3_Az** (a), **NO_2_-C_10_**∙∙∙**F_2_Az** (b) and **NO_2_-C_10_**∙∙∙**F_2_’Az** (c) showing the phase transition upon cooling from the isotropic melt (I) to the nematic (N) or crystalline phase (Cr).

The azo compounds with a weakly polarised iodine atom (**F_2_Az** or **F_2_’Az**) have significantly lower interaction energies and halogen bonding appears not sufficiently strong enough to extend the mesogenic core at elevated temperatures. Thus, no liquid crystalline phase was observed for the **NO_2_-C_10_**∙∙∙**F_2_Az** and **NO_2_-C_10_**∙∙∙**F_2_’Az** assemblies (see Figure 5). In contrast, the highly fluorinated azo compounds **F_4_Az** and **F_3_Az** with a stronger polarisation on the iodine atom form sufficiently strong halogen bonds with **NO_2_-C_10_** to extend the mesogenic core and induce liquid crystallinity (see Figure 6) [12].

It appears surprising, that **NO_2_-C_10_**∙∙∙**F_3_Az** shows a broader nematic mesophase range (Δ*T* = 12.5 °C) than **NO_2_-C_10_**∙∙∙**F_4_Az** (Δ*T* = 4.6 °C). However, the increase in the temperature range is predominantly attributed to the lowering of the crystallisation temperature, which indicates that the strength of the halogen bond is not the only contributor to the mesomorphic behaviour of the halogen-bonded materials. The change in the electronic anisotropy by unsymmetrical substitution with fluorine as present in **F_3_Az** will also have an impact on the dispersion interactions and packing in the solid state and adds to the shift of the crystallisation temperature.

### Photophysical studies

**Figure 7:**
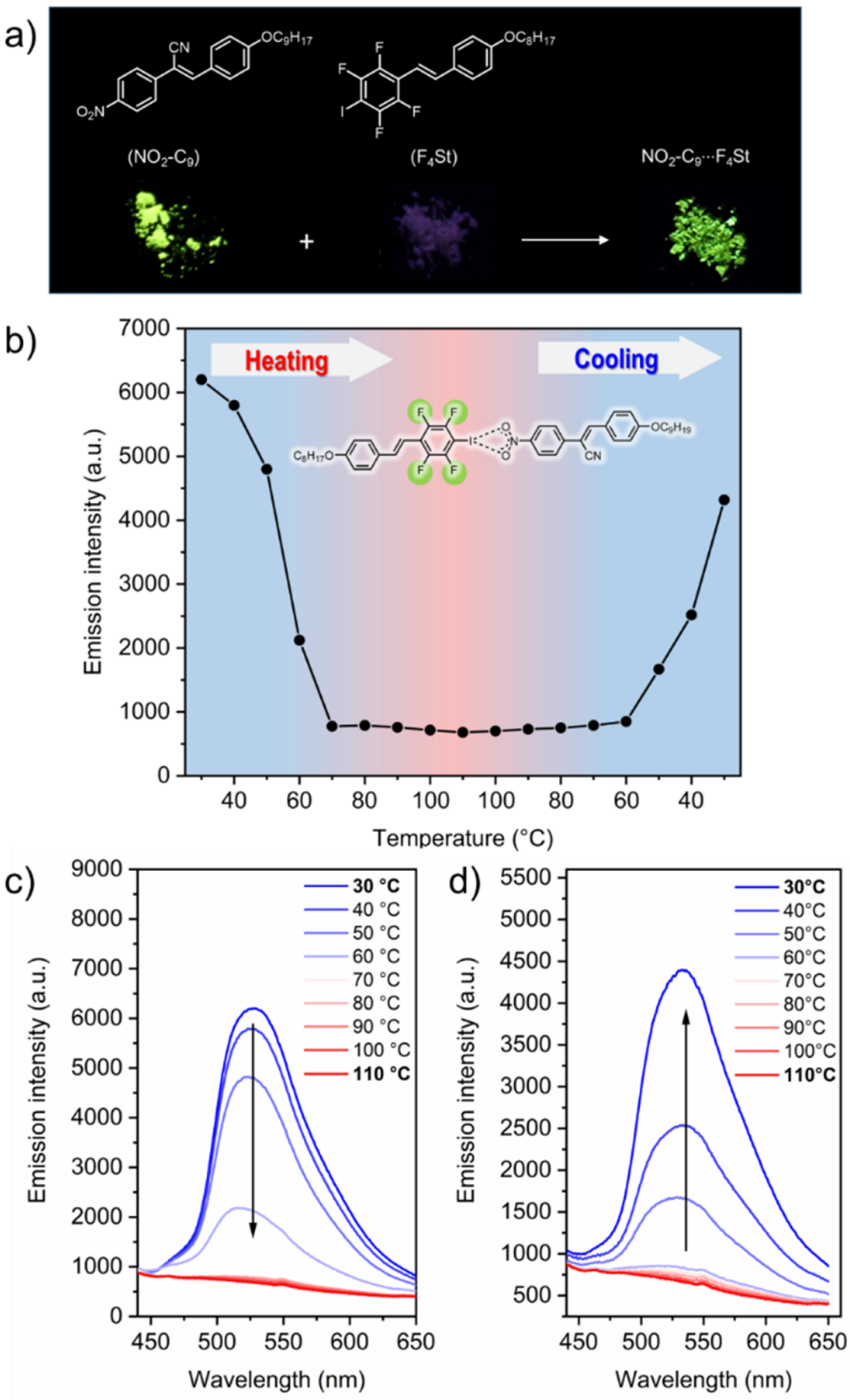
Fluorescence studies of **NO_2_-C_9_**∙∙∙**F_4_St**. The photographs of the solid components as well as the formed complex under UV light irradiation (λ_ex_ = 365 nm) reveal the change in fluorescent intensity upon complexation (a); fluorescence intensity plot as a function of the temperature upon heating from room temperature to 110 °C and subsequent cooling back to room temperature (b); emission spectra showing a gradual decrease in emission intensity upon heating (c) and cooling (d).

Recently, our group has shown that self-assembly provides an efficient way to tune fluorescence behaviour of liquid crystalline materials [21]. Phenolic thioethers showing aggregation-induced emission properties were combined with alkoxystilbazoles to form hydrogen-bonded mesogens. Since **NO_2_-C*_n_*** is known to be fluorescent, we were curious how the formation of the halogen-bonded complexes affects the AIE behaviour of **NO_2_-C*_n_***. Therefore, we studied the photophysical properties of 1:1 assemblies by UV–vis and fluorescence spectroscopy. The **NO_2_-C*_n_*** building blocks are moderately fluorescent in the solid state and **F_4_St** as well as **F_4_Az** show no significant fluorescence. However, upon formation of the halogen-bonded assemblies the fluorescence of the materials is significantly changed. Since the **F_4_Az**-based assemblies did not show fluorescence behaviour, the following discussion focuses on the assemblies **NO_2_-C_9_**∙∙∙**F_4_St** as representative example. The fluorescence of **NO_2_-C_9_** appears yellow-green, while **F_4_St** is non-fluorescent. Upon formation of the halogen-bonded liquid crystal, green fluorescence was observed (see Figure 7).

**Figure 8:**
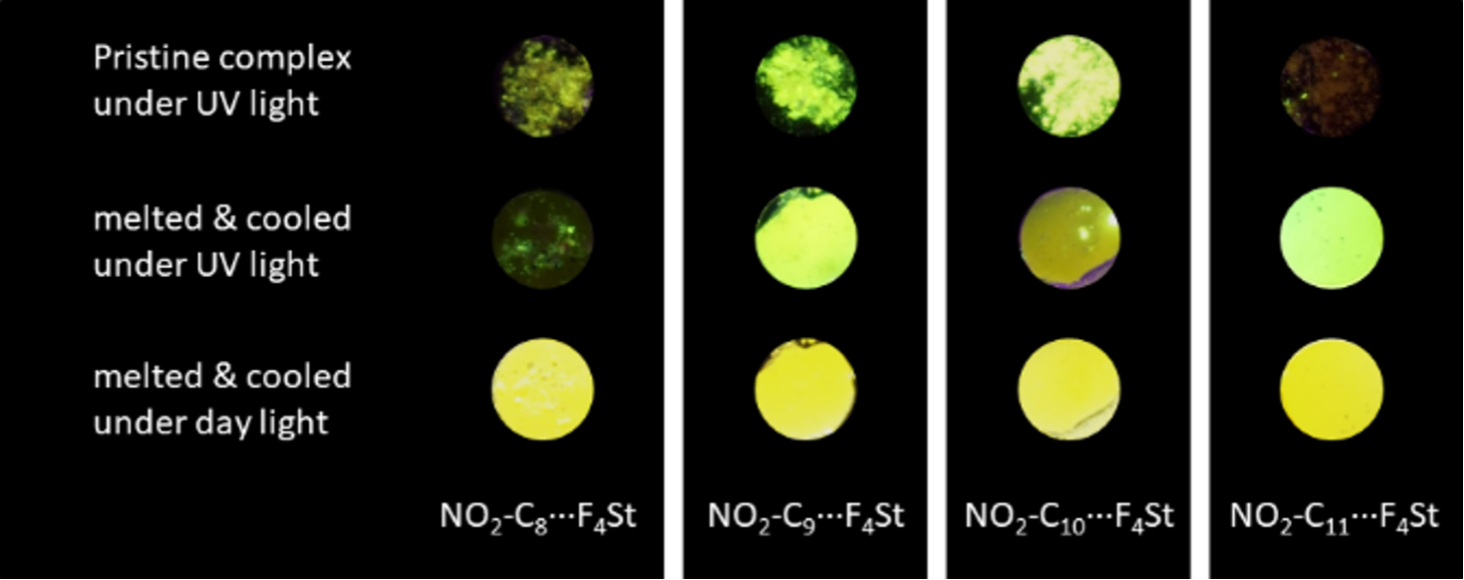
Photographs of the assemblies with different alkoxy chain lengths on the **NO_2_-C*_n_*** moiety directly after the removal of the solvent (pristine) and after one heating/cooling cycle reveal the impact of the alkoxy chain length and a significant rearrangement of the solid-state packing upon heating/cooling the sample (diameter of sample ≈ 0.8 cm).

Similar to the observation made for the liquid crystalline behaviour, also the fluorescence behaviour shows a strong odd–even effect for the alkoxy chain length at the **NO_2_-C*_n_*** unit, which indicates a significant difference in the molecular packing in the solid state. The fluorescence properties noticeably changed from the pristine assemblies directly after removal of the solvent, compared to the materials after one heating/cooling cycle (see Figure 8). In addition, the odd–even effect already described for the liquid crystalline properties also affects the fluorescence behaviour of the supramolecular assemblies in the solid state. Directly after removal of the solvent, all materials were obtained as crystalline powders with weak to moderate fluorescence as observed under UV light by the naked eye (see Figure 8). In contrast, after one heating/cooling cycle the fluorescence of the assemblies based on nitro-cyanostilbenes with an odd number of carbon atoms in the alkoxy chain significantly increased, while for the even-numbered **NO_2_-C*_n_*** no significant change in the fluorescence was observed.

The AIE behaviour of cyanostilbenes was first described by Park and co-workers [22]. This readily accessible class of fluorophores can easily be modified and attached to other functional entities, thus it has become a promising candidate for the design of new materials for optoelectronic applications [23]. Recently, it was successfully employed as a fluorescent moiety in the design of luminescent liquid crystals. In supramolecular liquid crystals, however, it did not find application so far [24].

**Figure 9:**
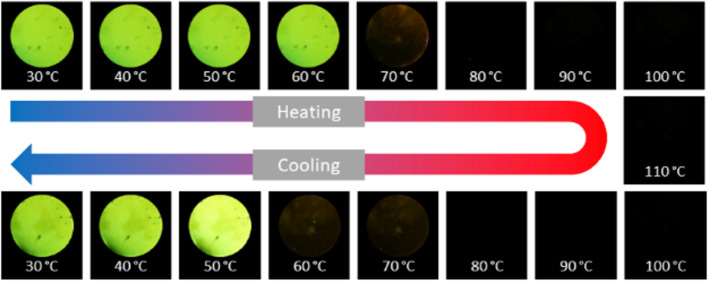
Temperature-dependent fluorescent images of **NO_2_-C_9_**∙∙∙**F_4_St** showing the enhancement of emission upon aggregation at room temperature (λ_ex_ = 365 nm).

We also studied the temperature dependence of the fluorescence behaviour. In the solid state at room temperature, **NO_2_-C_9_**∙∙∙**F_4_St** shows a high intensity emission at λ = 545 nm, which gradually decreases with increasing temperature. At 70 °C, the fluorescence is almost completely quenched. This temperature correlates with the transition from the crystalline phase to the liquid crystalline phase as observed by POM and DSC. The further heating to 110 °C did not yield significant changes in the fluorescence intensities (see Figure 9). Upon cooling the sample to room temperature, the fluorescence returned, which is in line with the previous reports and characteristic for mesogens with AIE behaviour [25].

## Conclusion

In summary, the first example of a halogen-bonded fluorescent liquid crystal is reported, employing the interaction between iodofluorobenzene derivatives and nitro-cyanostilbenes. A systematic investigation of the materials revealed the impact of the halogen bond donor on the liquid crystalline properties of the assemblies. While the stilbene-based halogen bond donor induces the formation of nematic mesophases with broad temperature ranges, the temperature range of the mesophase of the azobenzene-based assemblies is significant narrower. Theoretical calculations and the modular use of halogen bond donors with changing fluorination degree reveal that at least three fluorine atoms are needed for the formation of a thermally stable halogen bond to induce liquid crystalline properties.

In addition, the formation of the halogen-bonded assemblies had an impact on the fluorescence and photophysical properties of the supramolecular mesogens showing the characteristic AIE behaviour. The length of the terminal alkoxy chain at the nitro-cyanostilbene had a significant impact on the fluorescence behaviour, which was attributed to the packing differences of the assemblies with an even or an odd number of carbon atoms in the alkoxy chain. Currently we are preparing a comprehensive study investigating the potential of halogen bonding in fluorescent liquid crystalline materials.

## Supporting Information

File 1 Detailed descriptions of the experimental procedures and comprehensive analytical data.
