# Supplementary material for: Tuning the solid-state emission of liquid crystalline nitro-cyanostilbene by halogen bonding
Source: Beilstein J Org Chem. 2021 Jan 14;17:124–31. doi: 10.3762/bjoc.17.13 (PMC7814182; doi:10.3762/bjoc.17.13)
Supplement: File 1 — Detailed descriptions of the experimental procedures and comprehensive analytical data. [file Beilstein_J_Org_Chem-17-124-s001.pdf]

BEILSTEIN JOURNAL OF ORGANIC CHEMISTRY

Supporting Information

for

Tuning the solid-state emission of liquid crystalline nitro-cyanostilbene by halogen bonding

Subrata Nath, Alexander Kappelt, Matthias Spengler, Bibhisan Roy, Jens Voskuhl and Michael Giese

Beilstein J. Org. Chem. **2021,** *17,* 124–131. doi:10.3762/bjoc.17.13

Detailed descriptions of the experimental procedures and comprehensive analytical data

License and Terms: This is a supporting information file under the terms of the Creative Commons Attribution License (https://creativecommons.org/licenses/by/4.0). Please note that the reuse, redistribution and reproduction in particular requires that the author(s) and source are credited and that individual graphics may be subject to special legal provisions.
The license is subject to the *Beilstein Journal of Organic Chemistry* terms and conditions: (https://www.beilstein-journals.org/bjoc/terms)

1. Materials and methods

Compounds and solvents were used as obtained from suppliers without further purification. ^{1}H and ^{13}C NMR spectra of the intermediates and products were recorded in deuterated solvents ($CDCl_3$, DMSO-d_6 or MeOD) with a Bruker DRX 300. Mass spectra were obtained with a Bruker amaZon (MS) and IR spectra were recorded with a Varian 3100 FT-IR, Excalibur Series, ATR IR spectrometer. Polarized optical microscopy (POM) images/videos were taken on a Nikon Eclipse *Ni* equipped with an OptixCam Summit K2 OCS-D3K4-14-52X microscope camera and an LTS420 hotstage from Linkam Scientific Instruments Ltd. The images were recorded by an Imaging Source camera OptixCam K2. DSC thermograms were recorded using a Mettler Toledo DCS3+/700/866/Argon with a heating/cooling speed of 10 °C/min (sample weight ≈5 mg). UV–visible spectroscopy was performed using a Thermo Fisher Evolution 201 spectrophotometer.

Experimental procedures

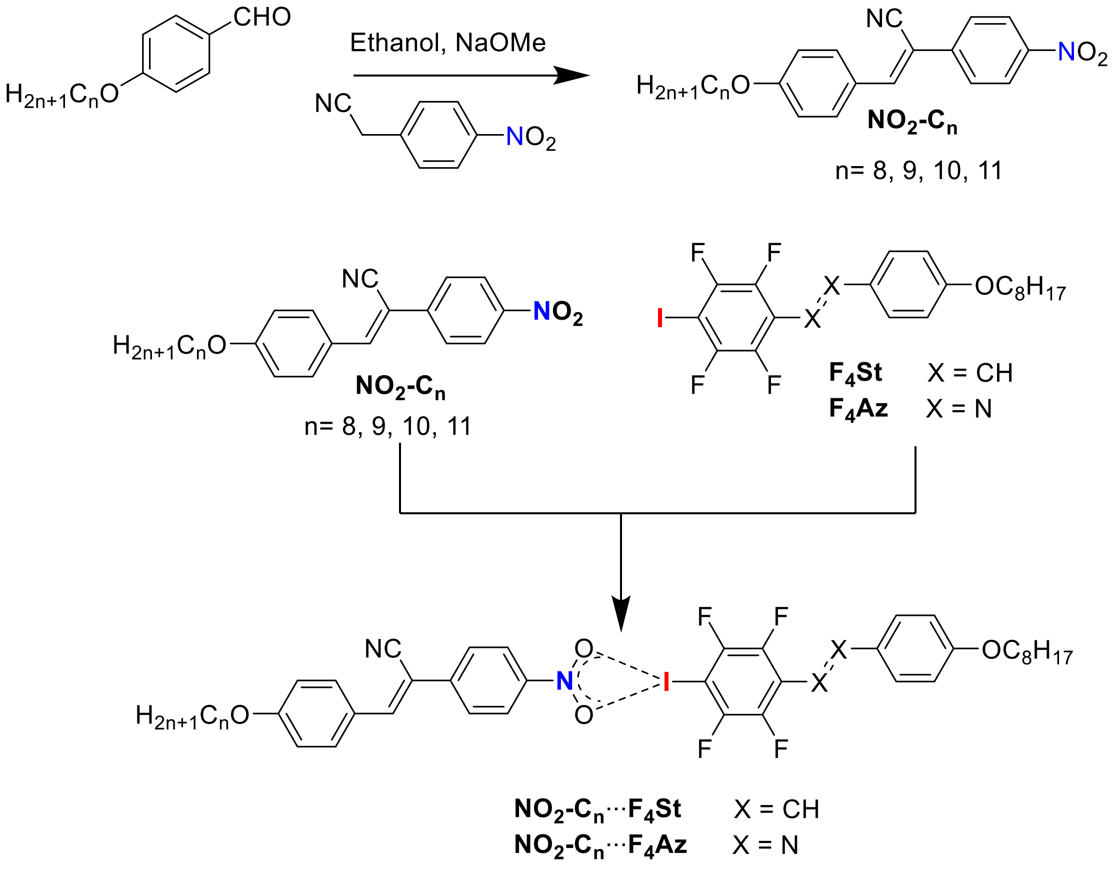

Scheme S1: The synthetic route for the synthesis of XB-acceptors, XB-donors and supramolecular assemblies.

Synthesis of compound NO_2-C_n (*n* = 1, 8, 9, 10, 11):

The desired compounds were synthesised based on a reported procedure[S1] with a small modification. 4-Alkoxybenzaldehyde (1 equiv), CH_3ONa (1 equiv) and 20 ml ethanol were placed into a 250 ml boiling flask. When the 4-alkoxybenzaldehyde and CH_3ONa had completely dissolved in ethanol, 4-nitrophenylacetonitrile (1 equiv) was added and the

mixture was stirred at 25 °C for 8 h. The final reaction mixture was filtered, the solid products were collected and washed several times with ethanol to obtain the yellow green solid products with a yield of 60–65%.

NO_2-C_1: ^{1}H NMR ($CDCl_3$, 300 MHz, ppm): δ 8.30-8.26 (m, 2H), 7.96 (d, *J* = 9Hz, 2H), 7.83-7.80 (m, 2H), 7.61 (s, 1H), 7.02 (d, *J* = 9Hz, 2H), 3.89 (s, 3H, 1×CH_3); ^{13}C ($CDCl_{3,}$ 75 MHz, ppm): δ 162.6, 147.7, 145.2, 141.3, 132.1, 126.5, 125.9, 124.5, 117.9, 114.8, 106.4, 55.7.
FT-IR (ATR): n (cm^{-1}) = 2978, 2843, 2212, 1597, 1579, 1508, 1381, 1334, 1311, 1261, 1180, 1034, 922, 848, 825, 752, 686, 621.
HRMS m/z (%): positive: calc. (M+Na^+): $C_{16}H_{12}N_2NaO_3^+$ = 303.0740, found: 303.0749.

NO_2-C_8: ^{1}H NMR ($CDCl_3$, 300 MHz, ppm): δ 8.31-8.28 (m, 2H), 7.94 (d, *J* = 9Hz, 2H), 7.83-7.80 (m, 2H), 7.60 (s, 1H), 7.0 (d, *J* = 9Hz, 2H), 4.04 (t, *J* = 6 Hz, 2H, OCH_2), 1.84-1.77 (m, 2H, 1×CH_2), 1.54-1.30 (m, 10H, 5×CH_2), 0.89 (t, *J* = 6Hz, 3H, 1×CH_3); ^{13}C NMR ($CDCl_{3,}$ 75 MHz, ppm): δ 161.5, 146.7, 140.2, 132.0, 130.3, 126.6, 125.1, 124.6, 115.4, 115.1, 109.3, 68.5, 32.0, 29.5, 29.4, 29.3, 26.2, 22.9, 14.3.
FT-IR (ATR): n (cm^{-1}) = 2962. 2920, 2854, 2212, 1578, 1508, 1333, 1311, 1259, 1198, 1180, 1110, 1039, 997, 914, 850, 825, 752, 688, 629.
HRMS m/z (%): positive: calc. (M+Na^+): $C_{23}H_{26}N_2NaO_3^+$: 401.1836, found: 401.1844.

NO_2-C_9: ^{1}H NMR ($CDCl_3$, 300 MHz, ppm): δ 8.30-8.27 (m, 2H), 7.94 (d, *J* = 9Hz, 2H), 7.84-7.80 (m, 2H), 7.60 (s, 1H), 7.01-6.98 (m, 2H), 4.04 (t, *J* = 6 Hz, 2H, OCH_2), 1.86-1.77 (m, 2H, 1×CH_2), 1.52-1.29 (m, 12H, 6×CH_2), 0.89 (t, J = 6Hz, 3H, 1×CH_3); ^{13}C NMR ($CDCl_{3,}$ 75 MHz, ppm): δ 162.3, 147.7, 145.3, 141.4, 132.2, 126.6, 125.7, 124.6, 118.0, 115.4, 106.2, 68.6, 32.1, 29.7, 29.6, 29.5, 29.3, 26.2, 22.9, 14.3.
FT-IR (ATR): n (cm^{-1}) = 2920, 2852, 2214, 1575, 1508, 1472, 1375, 1335, 1311, 1263, 1197, 1177, 1108, 1015, 917, 880, 849, 832, 751, 687, 623.
HRMS m/z (%): positive: calc. (M+Na^+): $C_{24}H_{28}N_2NaO_3^+$: 415.1992, found: 415.2003.

NO_2-C_{10}: ^{1}H NMR ($CDCl_3$, 300 MHz, ppm): δ 8.29 (d, *J* = 9Hz, 2H) 7.94 (d, *J* = 9Hz, 2H), 7.94-7.79 (m, 2H), 7.60 (s, 1H), 6.99 (d, 2H), 4.04 (t, *J* = 6 Hz, 2H, OCH_2), 1.86-1.77 (m, 2H, 1×CH_2), 1.47-1.29 (m, 14H, 7×CH_2), 0.89 (t, *J* = 6Hz, 3H, 1×CH_3); ^{13}C NMR ($CDCl_{3,}$ 75 MHz, ppm): δ 162.3, 147.7, 145.3, 141.4, 132.2, 126.5, 125.7, 124.5, 118.0, 115.3, 106.1, 68.6, 32.1, 29.8, 29.6, 29.5, 29.3, 26.2, 22.9, 14.3.
FT-IR (ATR): n (cm^{-1}) = 2960, 2918, 2850, 2210, 1599, 1578, 1506, 1469, 1333, 1309, 1255, 1179, 1107, 1047, 1020, 993, 914, 850, 823, 796, 752, 717, 688, 607.
HRMS m/z (%): positive: calc. (M+Na^+): $C_{25}H_{30}N_2NaO_3^+$: 429.2149, found: 429.2157.

NO_2-C_{11}: ^{1}H NMR ($CDCl_3$, 300 MHz, ppm): δ 8.30-8.27 (m, 2H), 7.94 (d, *J* = 9Hz, 2H), 7.84-7.80 (m, 2H), 7.60 (s, 1H), 7.02-6.98 (m, 2H), 4.04 (t, *J* = 6 Hz, 2H, OCH_2), 1.84-1.79 (m, 2H, 1×CH_2), 1.47-1.29 (m, 16H, 8×CH_2), 0.91-0.86 (m, 3H, 1×CH_3); ^{13}C NMR ($CDCl_{3,}$ 75 MHz, ppm): δ 162.3,

147.5, 145.3, 141.4, 132.2, 126.5, 125.7, 124.5, 118.0, 115.4, 106.2, 68.6, 32.1, 29.8, 29.8, 29.7, 29.6, 29.5, 29.3, 26.2, 22.9, 14.3.

FT-IR (ATR): n (cm^{-1}) = 2916, 2850, 2216, 1604, 1585, 1516, 1465, 1433, 1379, 1340, 1309, 1271, 1178, 1115, 1066, 1026, 989, 945, 918, 881, 847, 825, 750, 719, 684, 627.

HRMS m/z (%): positive: calc. ($M+Na^+$): $C_{26}H_{32}N_2NaO_3^+$: 443.2305, found: 443.2316.

Synthesis of the complexes NO_2-C_n···F_4St & NO_2-C_n···F_4Az:

CH_2Cl_2 solutions of compound **NO_2-C_n** and compound **F_4St/F_4Az** [S2,S3] were mixed in a 1:1 molar ratio. The solvent was allowed to evaporate overnight followed by subsequent drying under vacuum to give an orange solid.

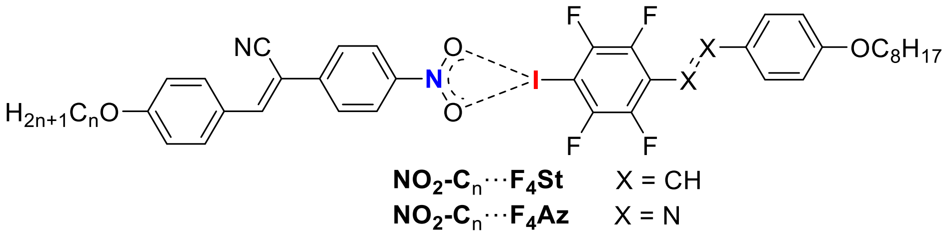

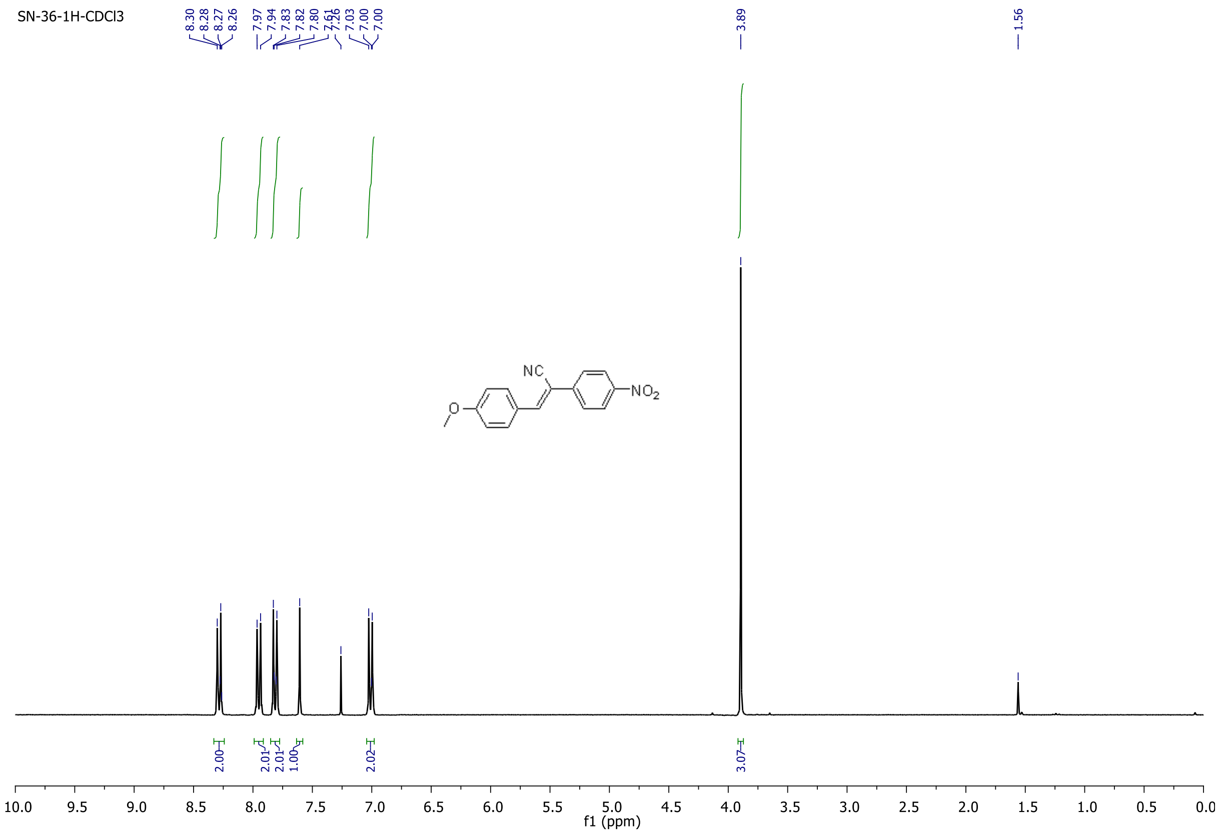

Figure S1: ^{1}H NMR (300 MHz) spectrum of **NO_2-C_1** in $CDCl_3$.

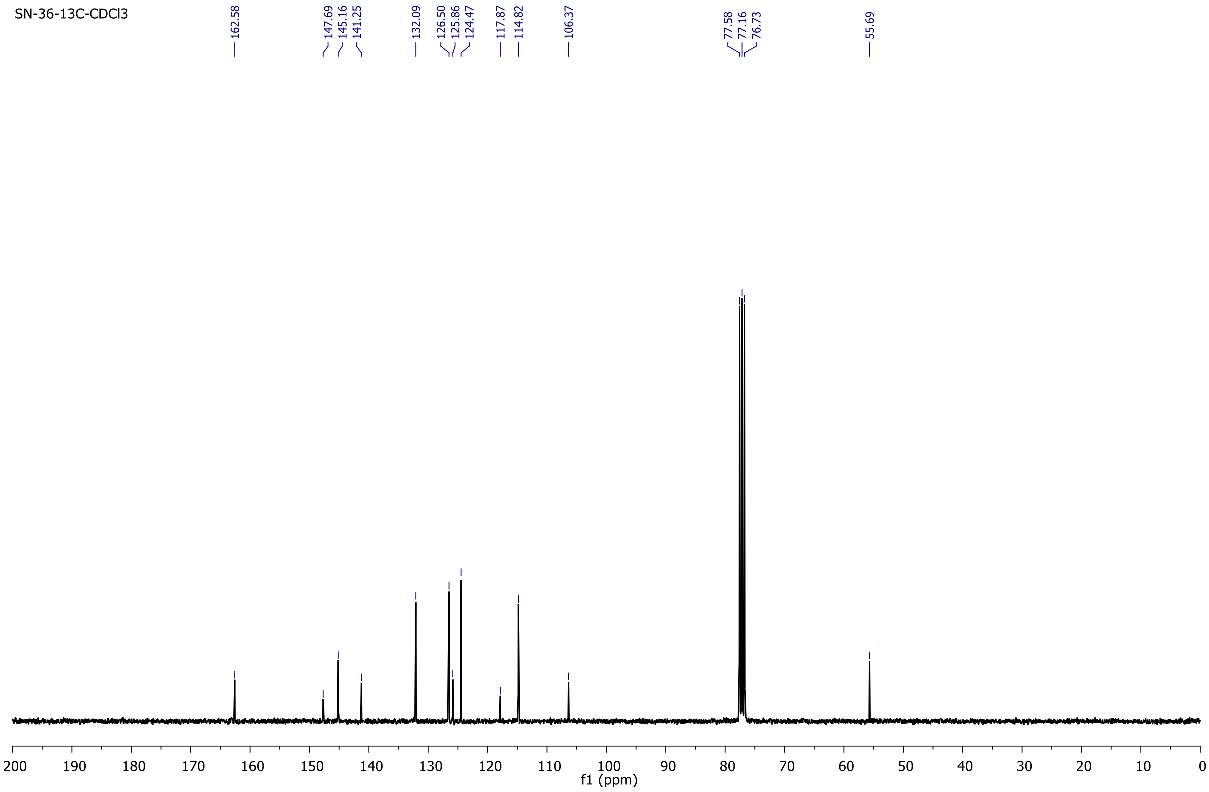

Figure S2: ^{13}C NMR (75 MHz) spectrum of **NO_2-C_1** in $CDCl_3$.

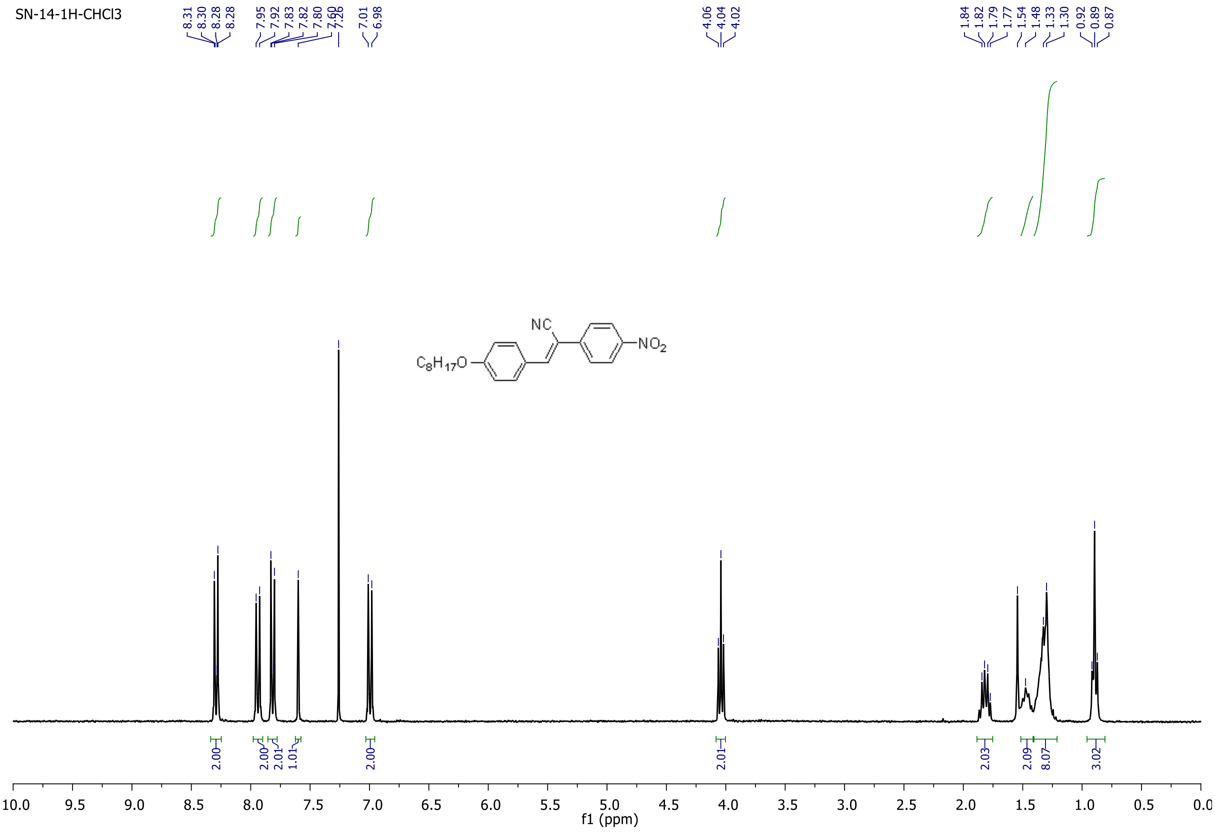

Figure S3: ^{1}H NMR (300 MHz) spectrum of **NO_2-C_8** in $CDCl_3$.

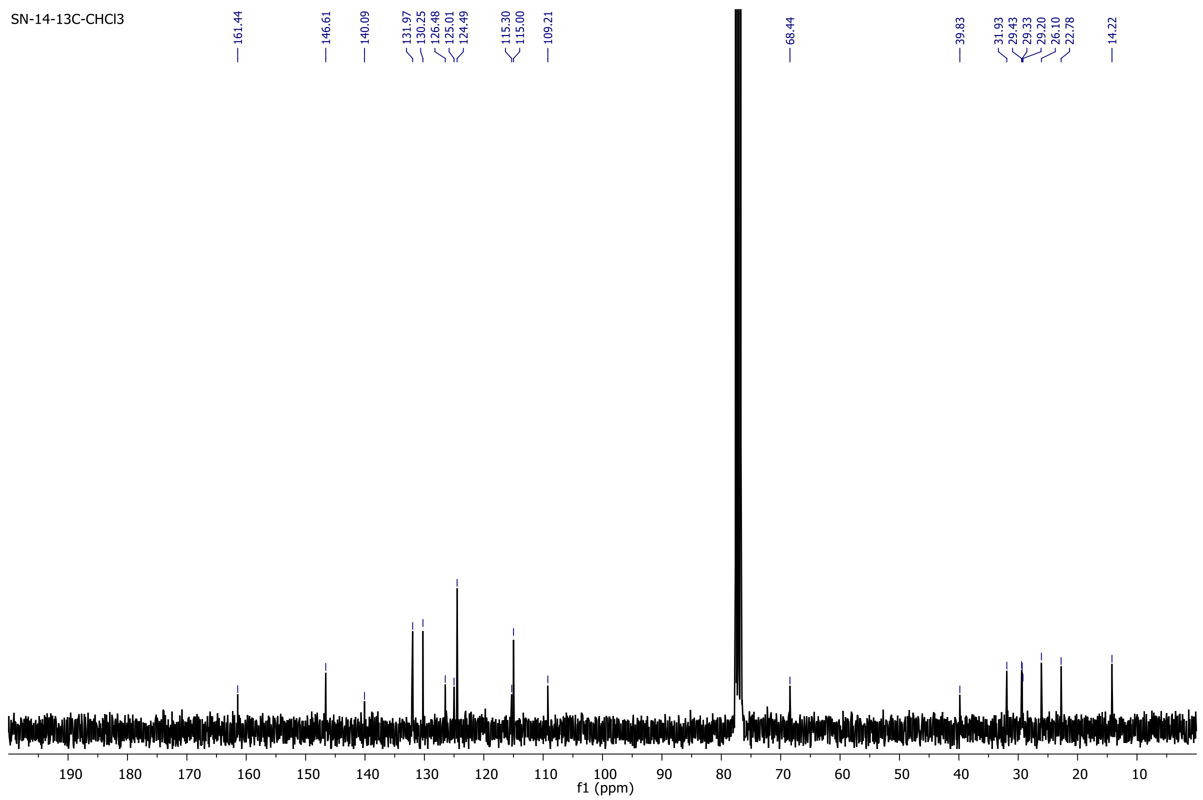

Figure S4: ^{13}C NMR (75 MHz) spectrum of **NO_2-C_8** in $CDCl_3$.

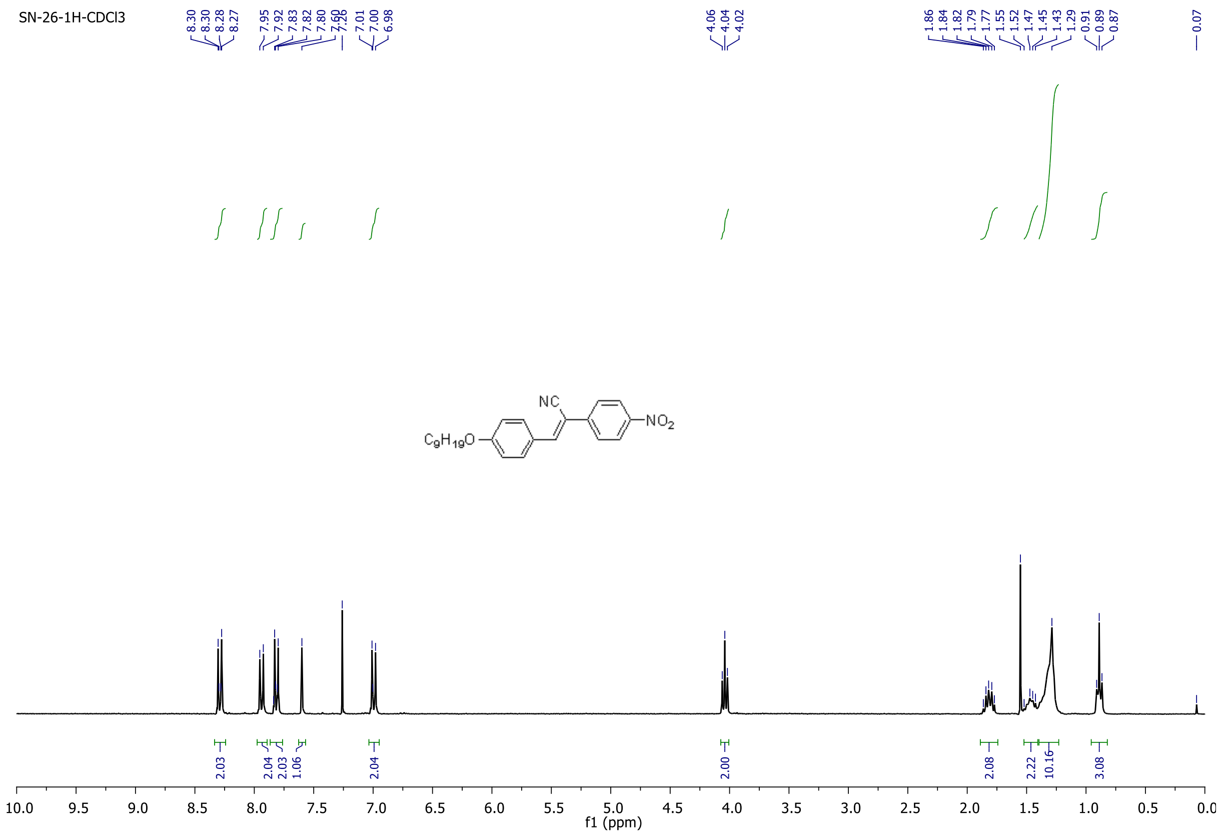

Figure S5: ^{1}H NMR (300 MHz) spectrum of **NO_2-C_9** in $CDCl_3$.

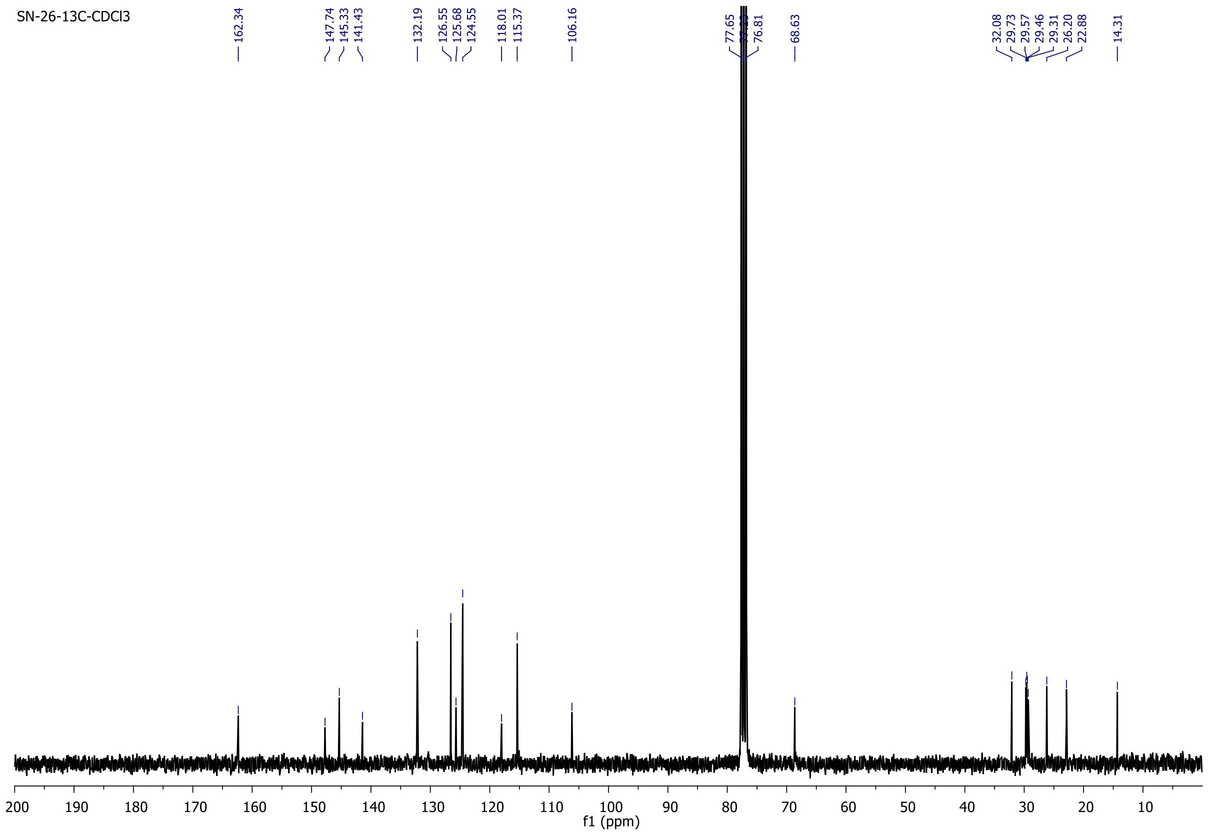

Figure S6: ^{13}C NMR (75 MHz) spectrum of **NO_2-C_9** in $CDCl_3$.

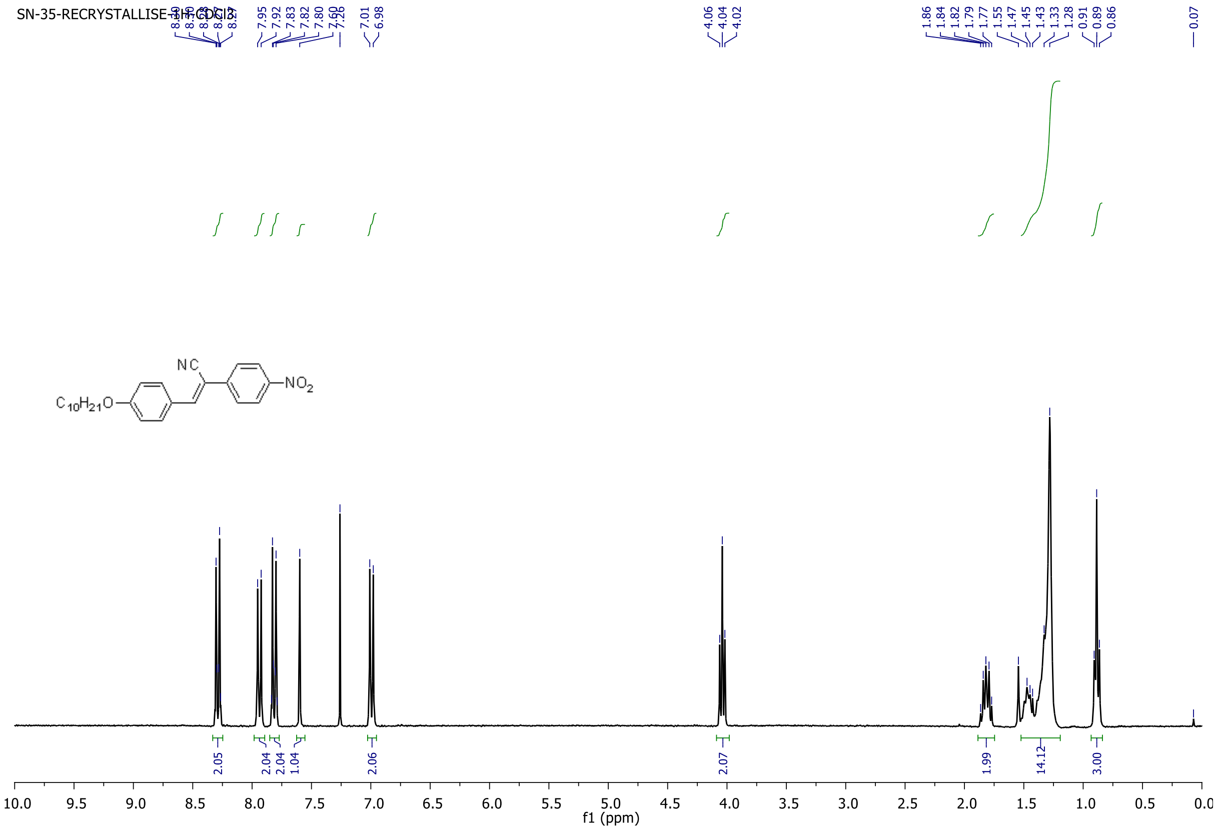

Figure S7: ^{1}H NMR (300 MHz) spectrum of **NO_2-C_{10}** in $CDCl_3$.

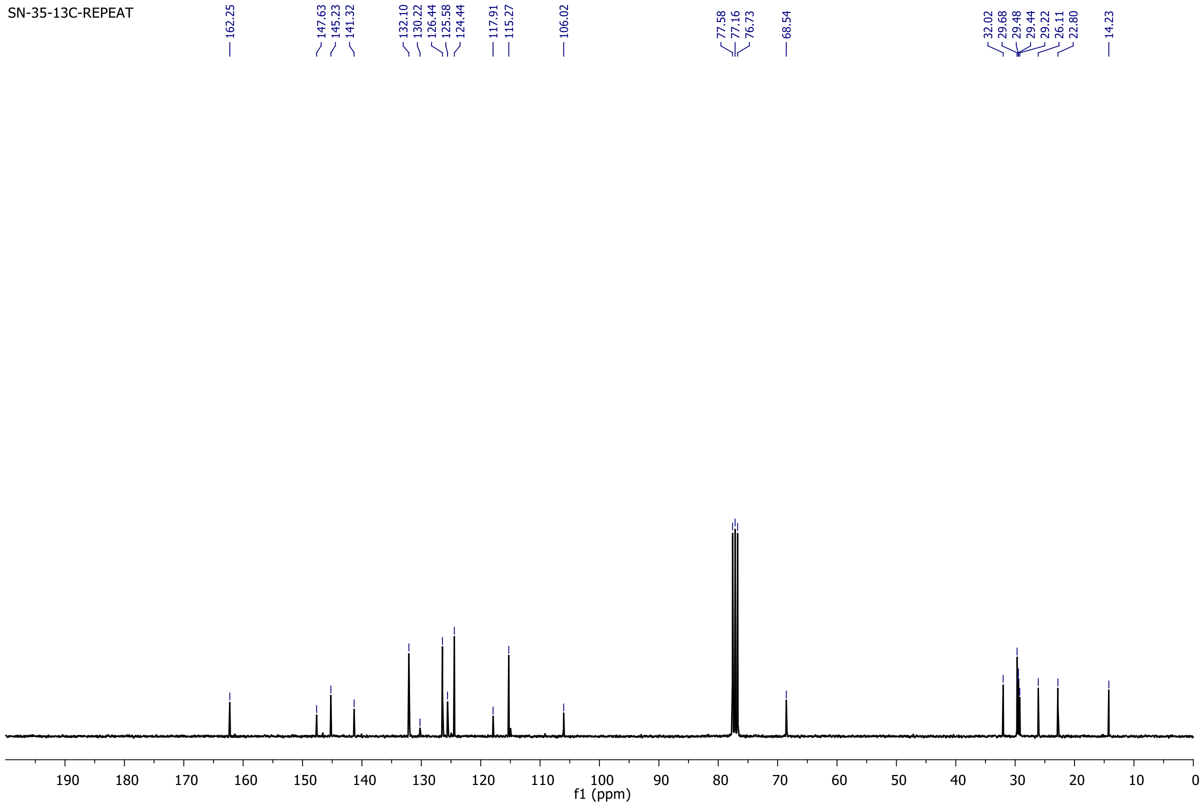

Figure S8: ^{13}C NMR (75 MHz) spectrum of **NO_2-C_{10}** in $CDCl_3$.

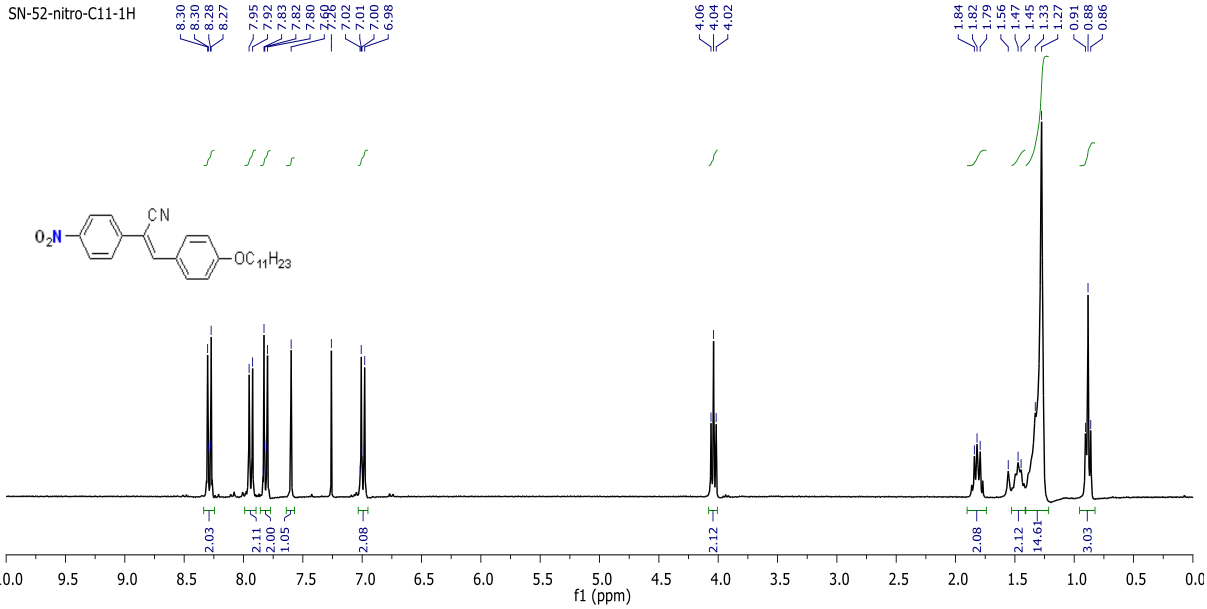

Figure S9: ^{1}H NMR (300 MHz) spectrum of **NO_2-C_{11}** in $CDCl_3$.

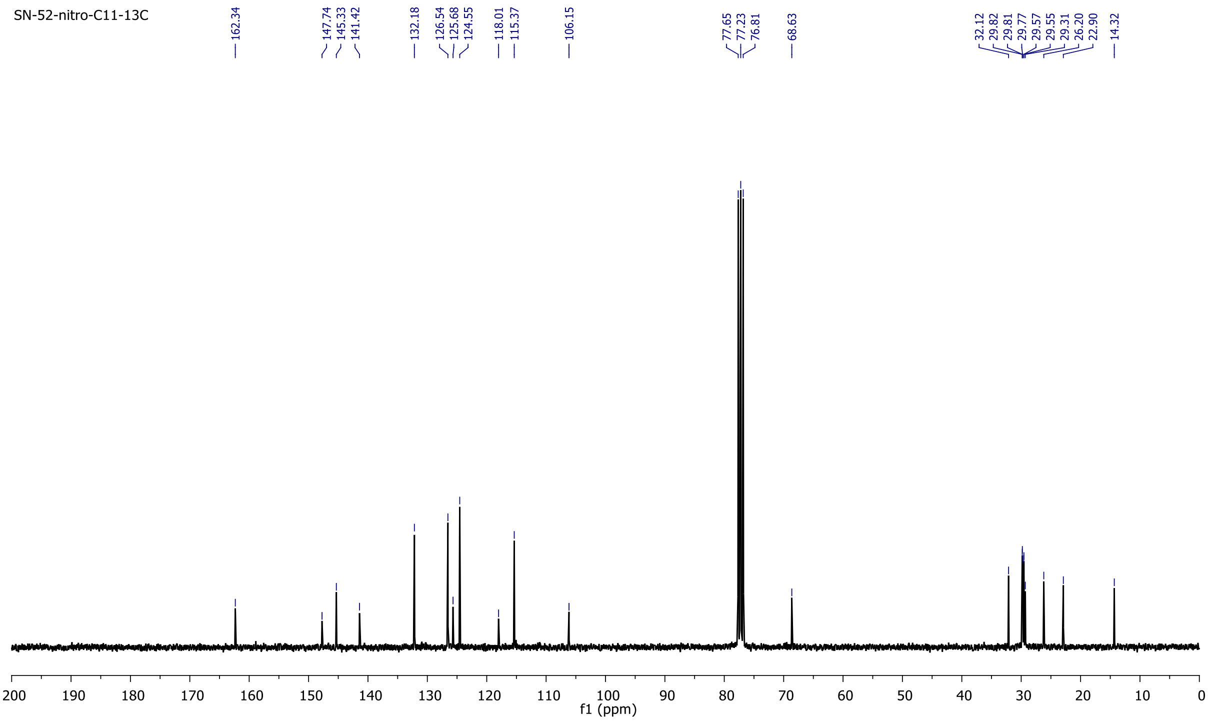

Figure S10: ^{13}C NMR (75 MHz) spectrum of **NO_2-C_{11}** in $CDCl_3$.

POM images:

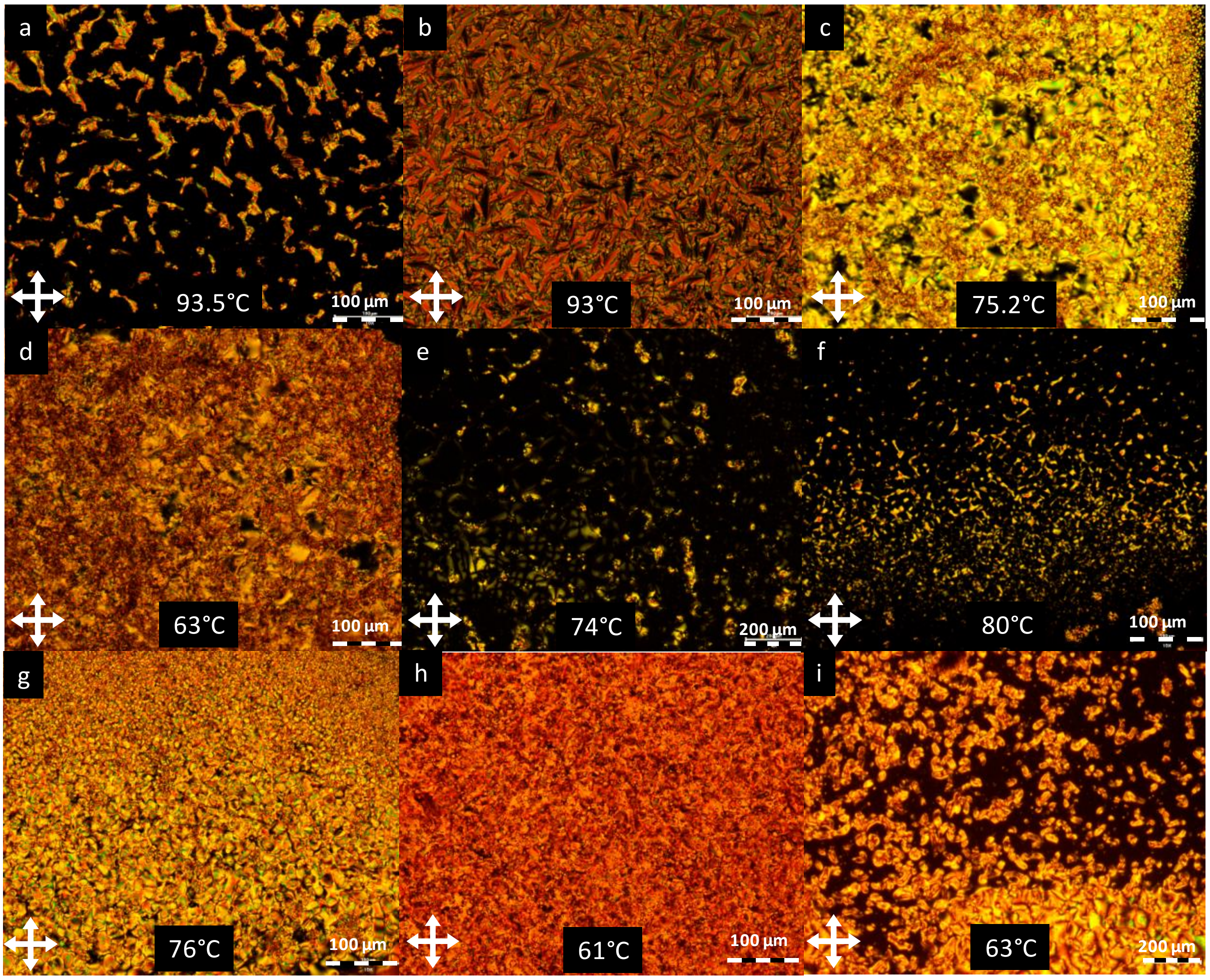

Figure S11: POM images of (a, b) **NO_2-C_{11}** (in heating and cooling, respectively); (c) **NO_2-C_8···F_4St** (cooling); (d) **NO_2-C_9···F_4St** (cooling); (e) **NO_2-C_{10}···F_4St** (cooling); (f, g) **NO_2-C_{11}···F_4St** (in heating and cooling, respectively); (h) **NO_2-C_9···F_4Az** (cooling) and (i) **NO_2-C_{11}···F_4Az** (cooling); at the different mentioned temperatures.

DSC thermograms:

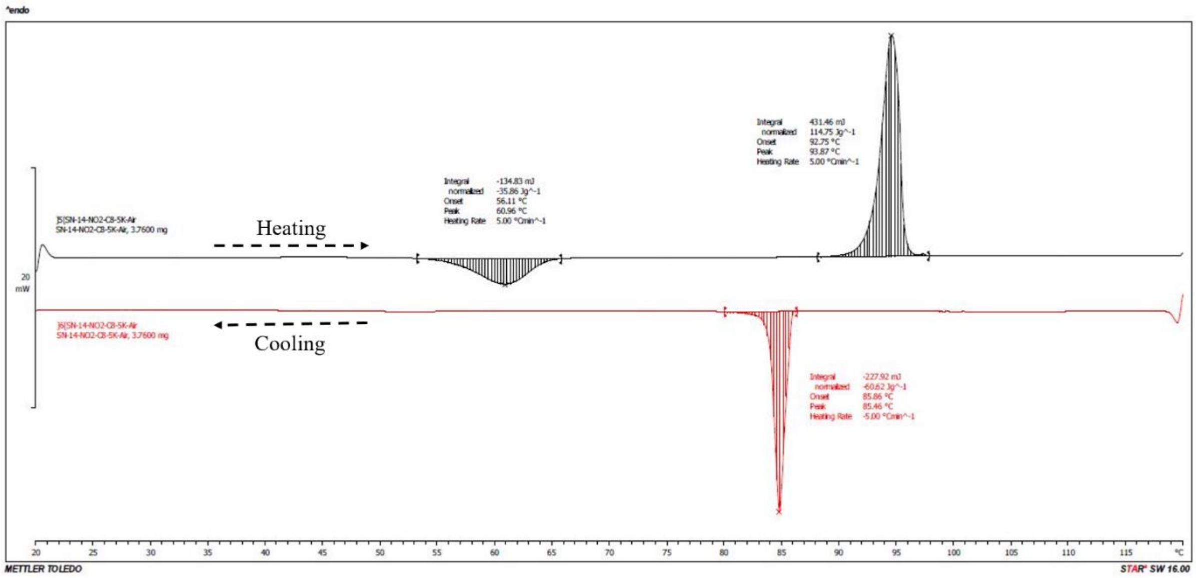

Figure S12: DSC traces obtained from the 2nd heating and 2nd cooling cycle at a rate of 5 °C min^{-1} for compound **NO_2-C_8**.

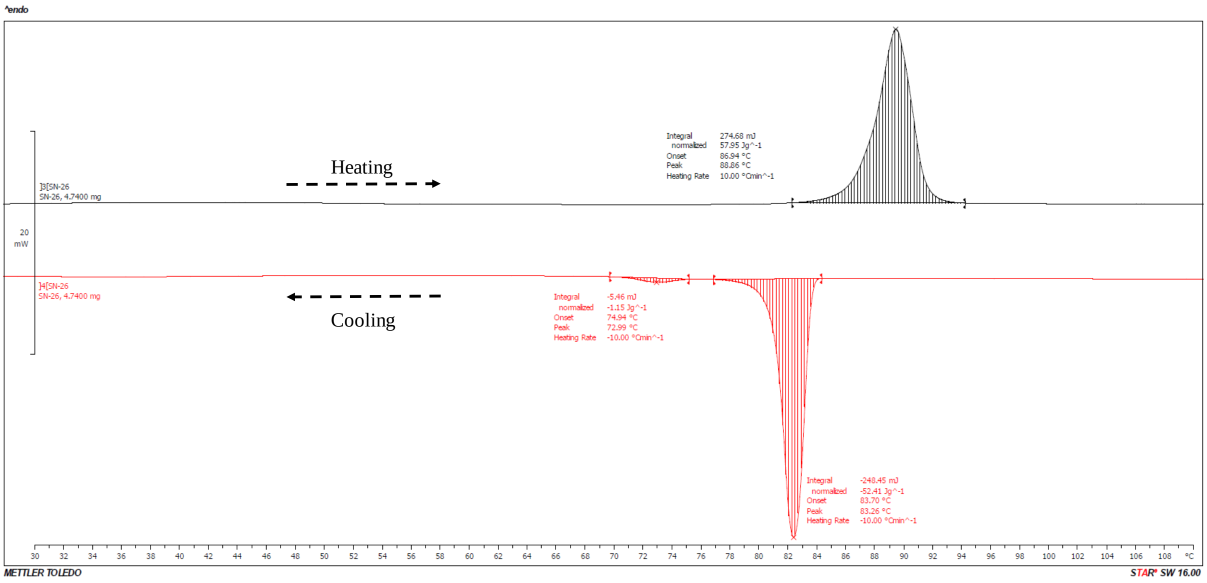

Figure S13: DSC traces obtained from the 2nd heating and 2nd cooling cycle at a rate of 10 °C min^{-1} for compound **NO_2-C_9**.

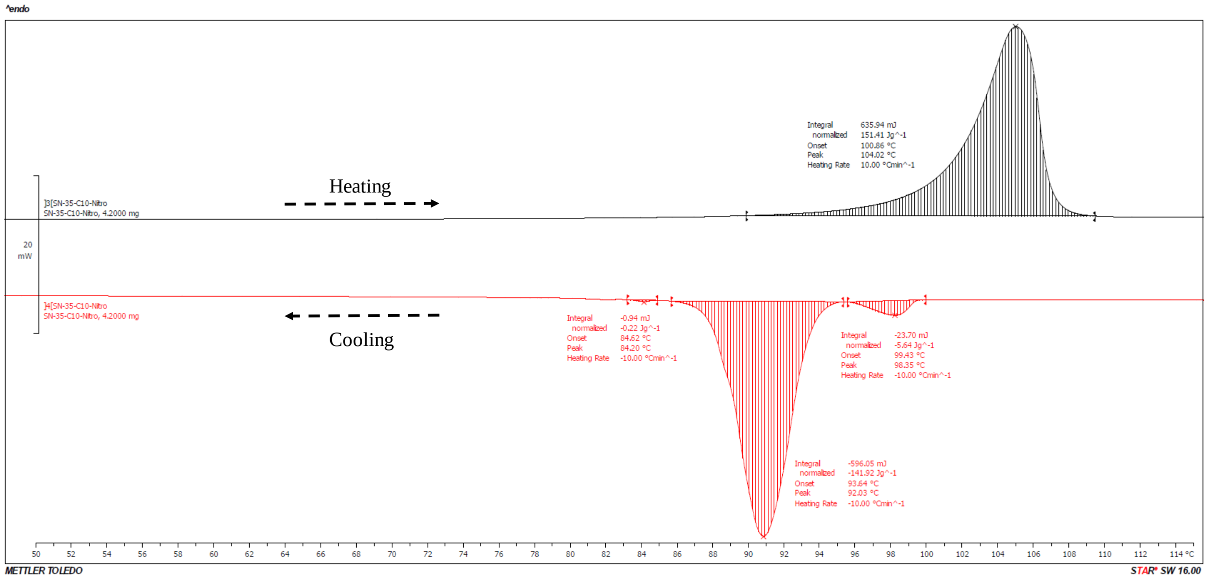

Figure S14: DSC traces obtained from the 2nd heating and 2nd cooling cycle at a rate of 10 °C min^{-1} for compound **NO_2-C_{10}**.

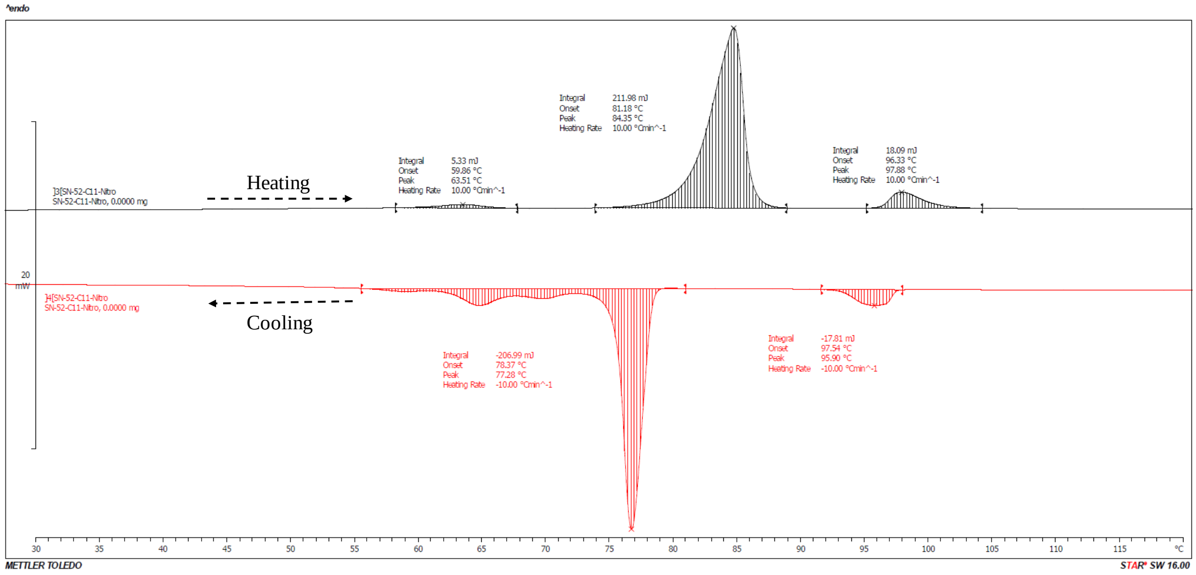

Figure S15: DSC traces obtained from the 2nd heating and 2nd cooling cycle at a rate of 10 °C min^{-1} for compound **NO_2-C_{11}**.

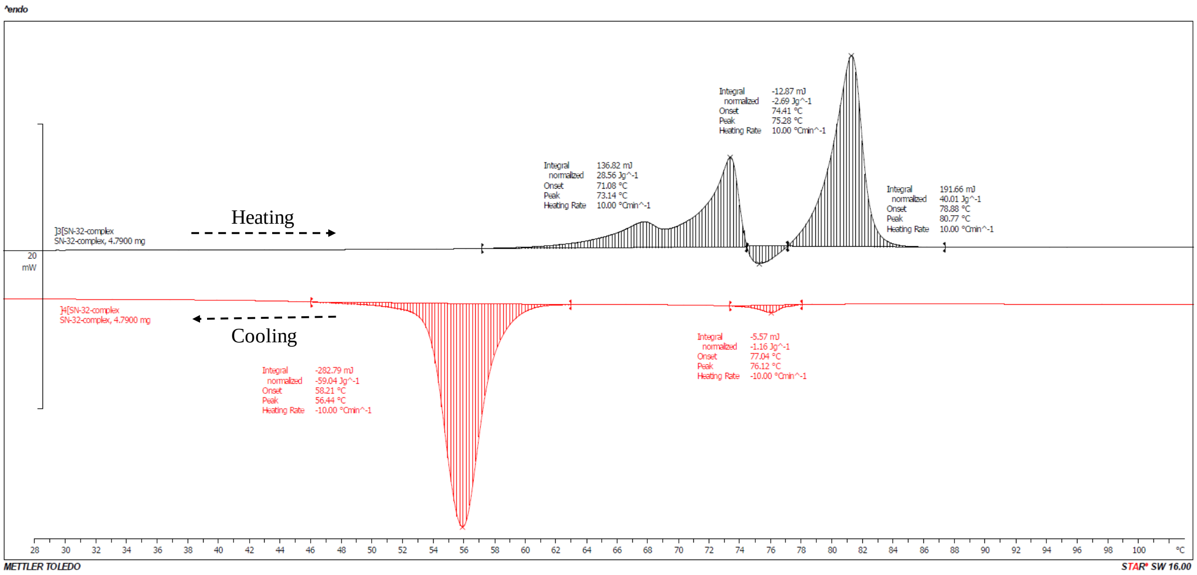

Figure S16: DSC traces obtained from the 2nd heating and 2nd cooling cycle at a rate of 10 °C min^{-1} for compound **NO_2-C_8···F_4St**.

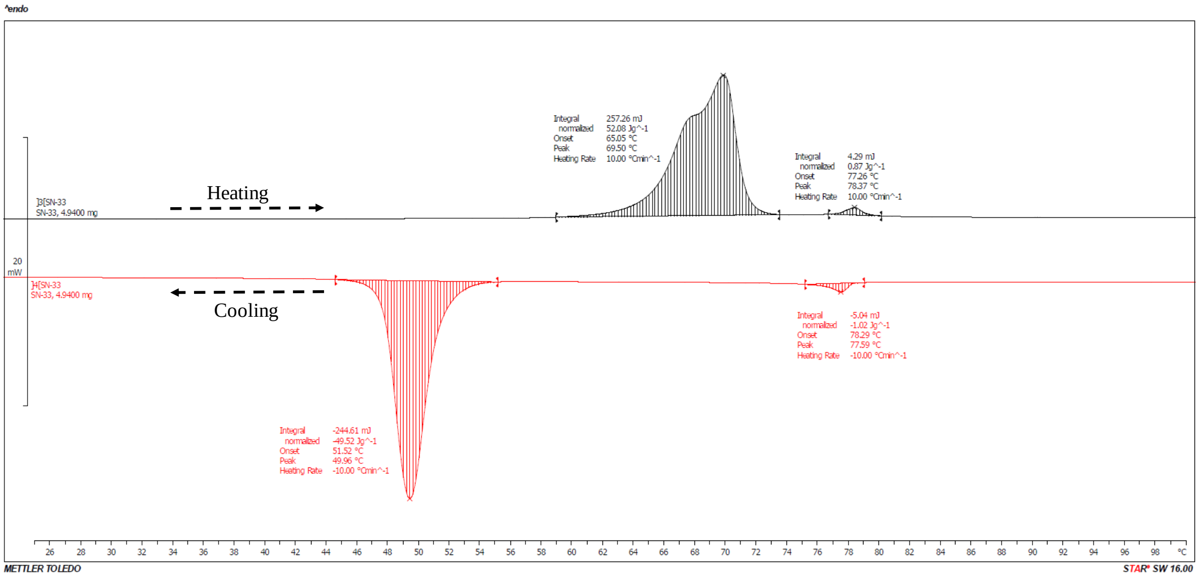

Figure S17: DSC traces obtained from the 2nd heating and 2nd cooling cycle at a rate of 10 °C min^{-1} for compound **NO_2-C_9···F_4St**.

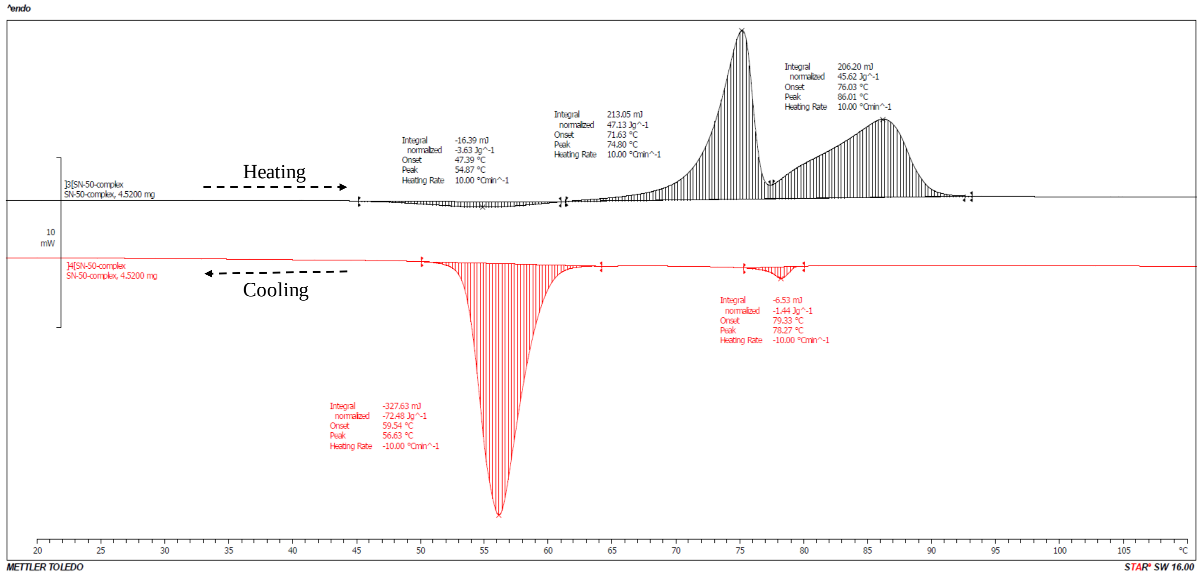

Figure S18: DSC traces obtained from the 2nd heating and 2nd cooling cycle at a rate of 10 °C min^{-1} for compound **NO_2-C_{10}⋯F_4St**.

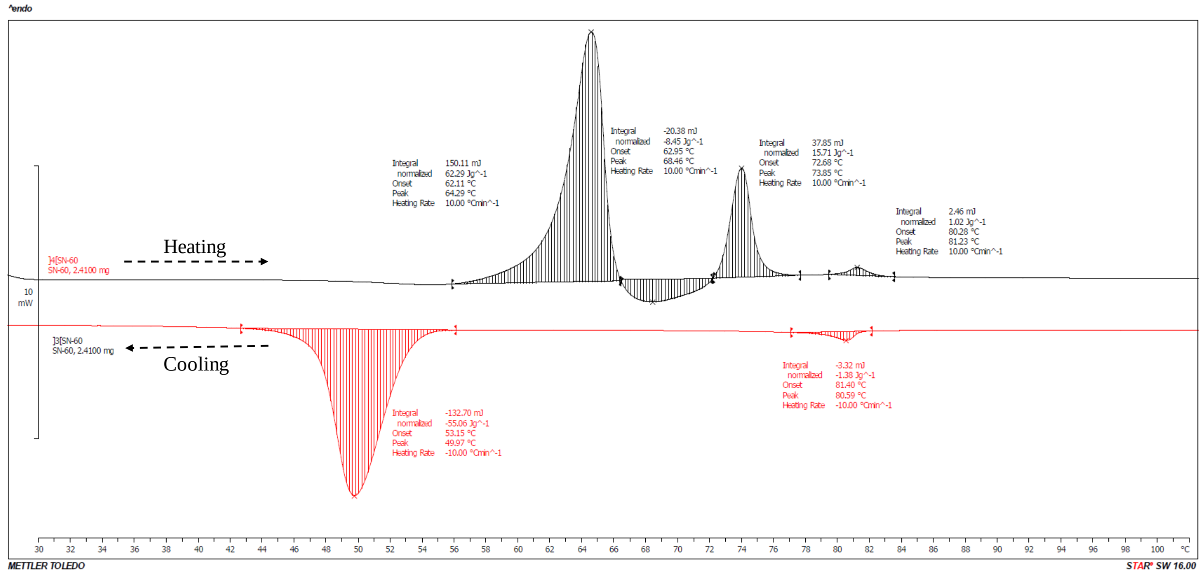

Figure S19: DSC traces obtained from the 2nd heating and 2nd cooling cycle at a rate of 10 °C min^{-1} for compound **NO_2-C_{11}⋯F_4St**.

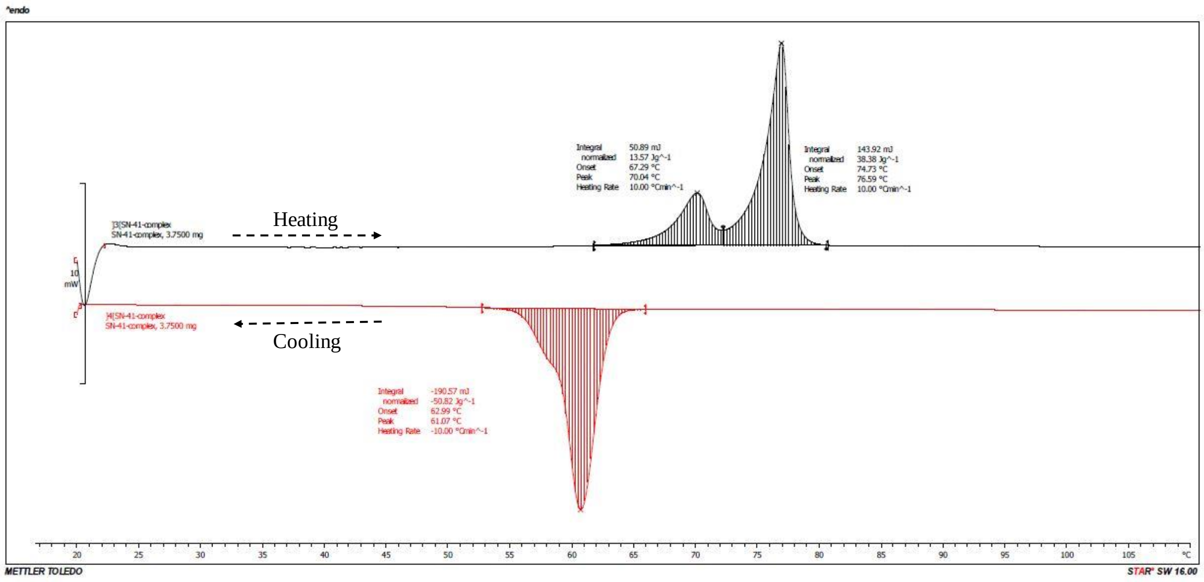

Figure S20: DSC traces obtained from the 2nd heating and 2nd cooling cycle at a rate of 10 °C min−1 for compound **NO_2-C_8···F_4Az**.

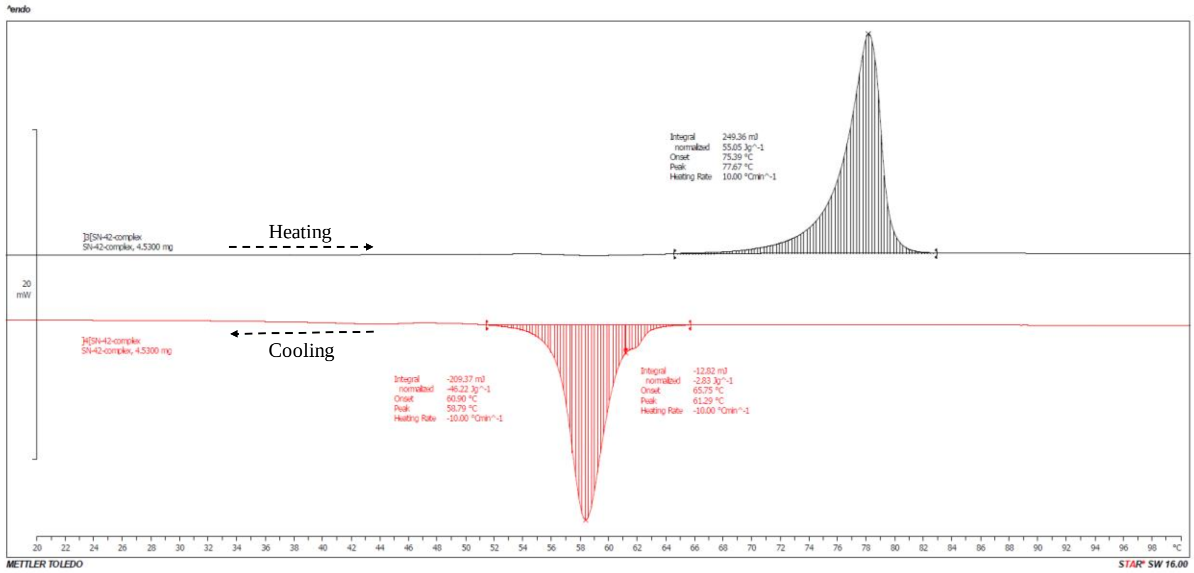

Figure S21: DSC traces obtained from the 2nd heating and 2nd cooling cycle at a rate of 10 °C min−1 for compound **NO_2-C_9···F_4Az**.

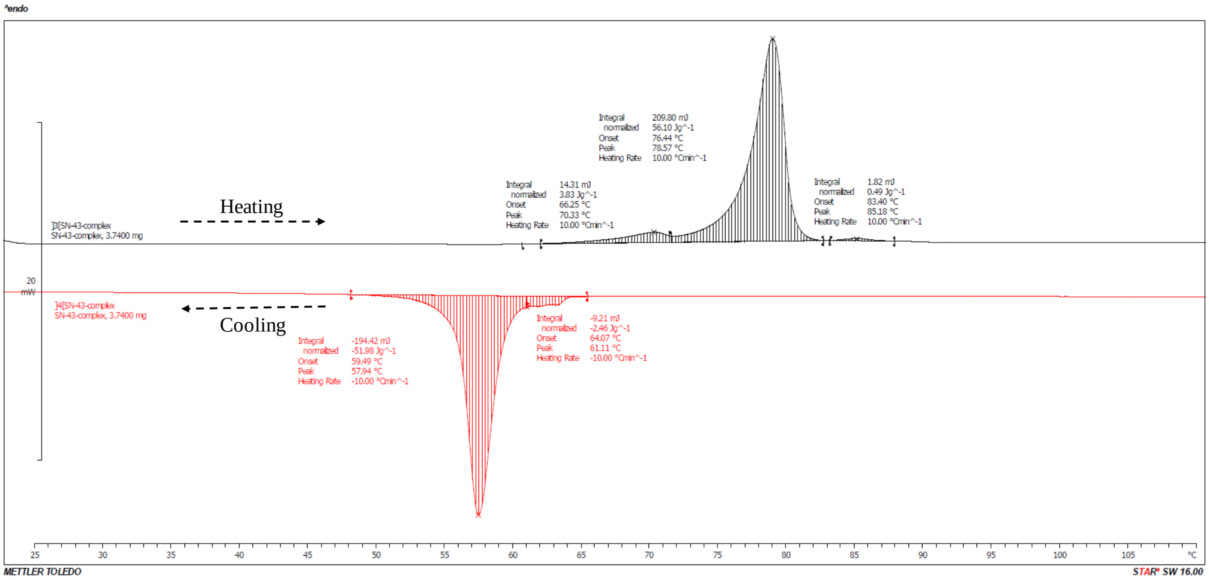

Figure S22: DSC traces obtained from the 2nd heating and 2nd cooling cycle at a rate of 10 °C min^{-1} for compound **NO_2-C_{10}···F_4Az**.

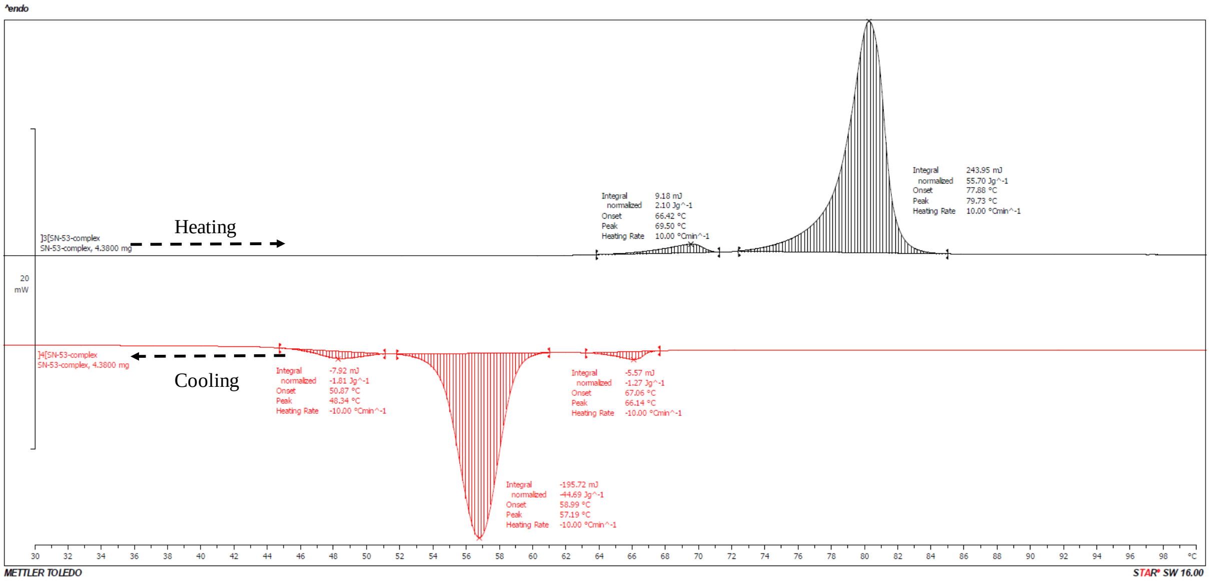

Figure S23: DSC traces obtained from the 2nd heating and 2nd cooling cycle at a rate of 10 °C min^{-1} for compound **NO_2-C_{11}···F_4Az**.

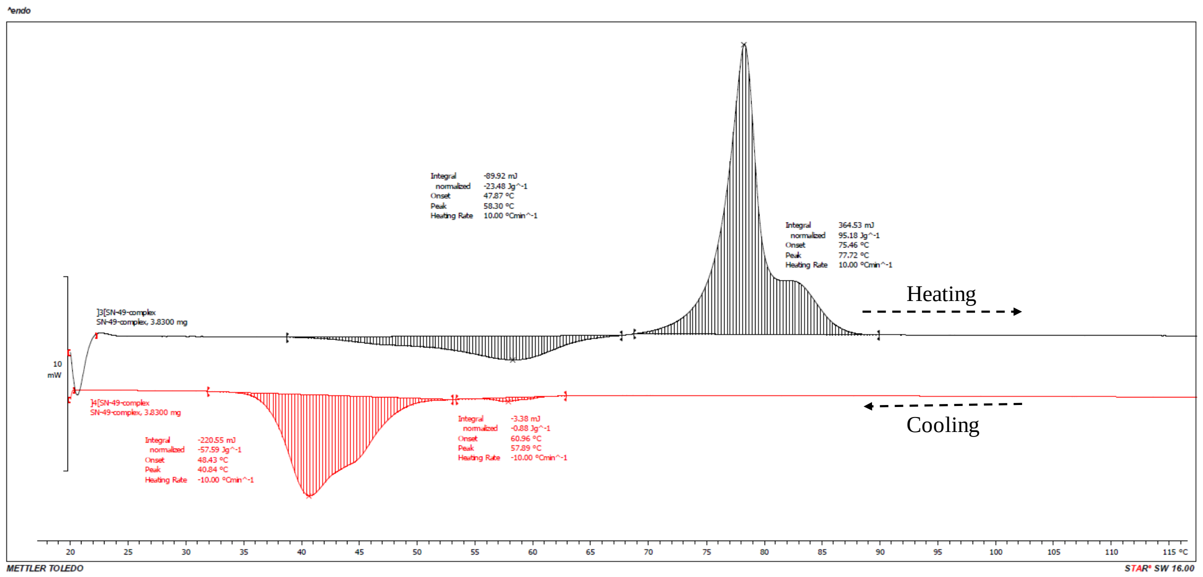

Figure S24: DSC traces obtained from the 2nd heating and 2nd cooling cycle at a rate of 10 °C min^{-1} for compound **NO_2-C_{10}···F_3Az**.

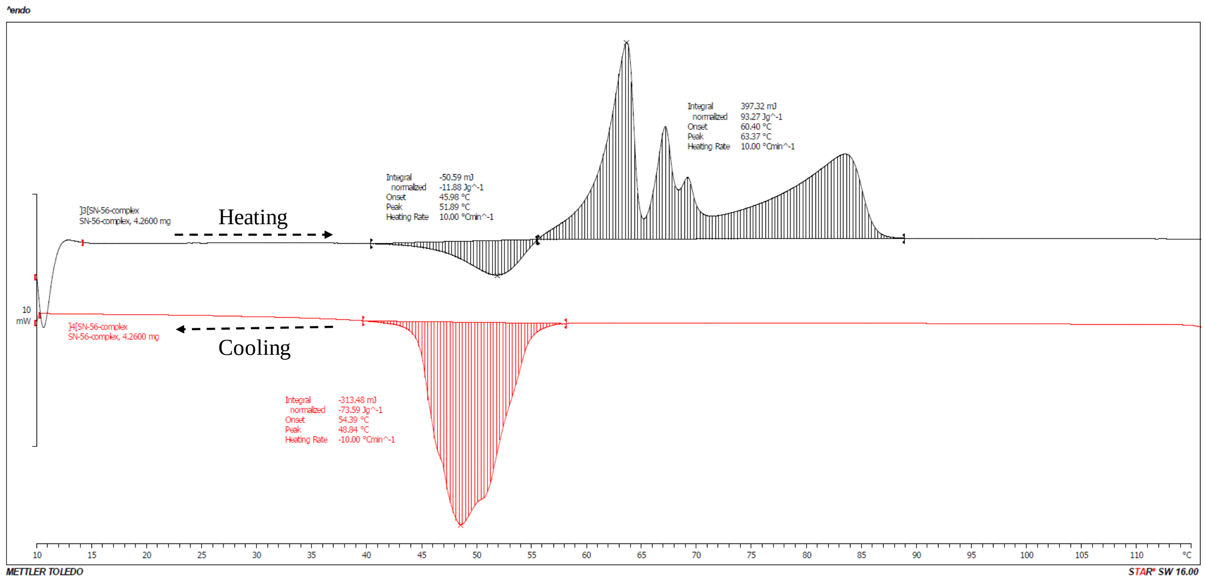

Figure S25: DSC traces obtained from the 2nd heating and 2nd cooling cycle at a rate of 10 °C min^{-1} for compound **NO_2-C_{10}···F_2Az**.

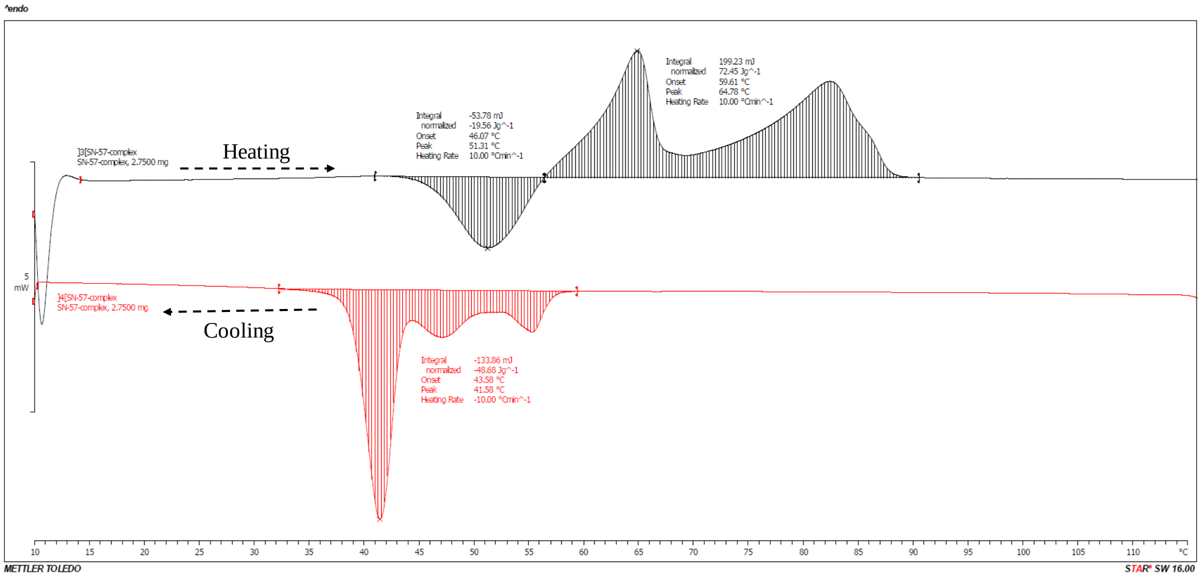

Figure S26: DSC traces obtained from the 2nd heating and 2nd cooling cycle at a rate of 10 °C min^{-1} for compound **NO_2-C_{10}···F_2′Az**.

Computational studies:

Theoretical specifications:

Structures of all complexes were optimised with the Gaussian 09 packages. The interaction energies were obtained as the energy differences counterpoise correction on the mp2/LanL2DZ. The basis set super position error (BSSE) has been undertaken for achieving errorless data of the supramolecule[S4,S5].

Imaginary modes were minimised using the screwing method. In this method, the optimised geometry is resubmitted upon manual displacement of the second frequency. During the second job submission, the command **Opt = tight** was used.

Theoretical specifications: p opt freq mp2/LanL2DZ counterpoise = 2

Table S1: Calculated interaction energies of the different complexes.

Complex	E_{AB} (BSSE corrected) *(Hartree)*	(E_A+E) BSSE Corrected *(Hartree)*	$E_{int.}$ (BSSE corrected) *(Hartree)*	$E_{int.}$*(BSSE corrected) (kJ/Mol)*
**NO_2-C_1···F_4St**	*-2006.44759*	*-2006.446575*	*-0.007692308*	*-20.19*
**NO_2-C_1···F_4Az**	*-2038.42375*	*-2038.421206*	*-0.006620833*	*-17.38*
**NO_2-C_1···F_3Az**	*-1939.4923*	*-1939.4893*	*-0.006201607*	*-16.28*
**NO_2-C_1···F_2Az**	*-1840.77384*	*-1840.766828*	*-0.00466*	*-12.23*
**NO_2-C_1···F_2′Az**	*-1840.75995*	*-1840.753543*	*-0.004406667*	*-11.57*

Coordinates for the optimised geometry of NO_2-C_1···F_4St

Charge = 0; multiplicity = 1 in supermolecule

Charge = 0; multiplicity = 1 in fragment 1.

Charge = 0; multiplicity = 1 in fragment 2.

C(Fragment=1)	-3.19669	2.82106	-0.01305
C(Fragment=1)	-2.01506	3.05855	-0.74136
C(Fragment=1)	-2.07746	3.71164	-1.9783
C(Fragment=1)	-3.27913	4.27811	-2.39692
C(Fragment=1)	-4.44271	4.09723	-1.65057
C(Fragment=1)	-4.43183	3.31767	-0.4797
C(Fragment=1)	-5.73348	4.8551	-2.10671
C(Fragment=1)	-6.97153	4.30557	-2.07845
C(Fragment=1)	-7.20384	2.78492	-2.00605
C(Fragment=1)	-8.51771	2.29513	-2.00811
C(Fragment=1)	-8.77283	0.96244	-1.99046
C(Fragment=1)	-7.72319	0.03958	-1.99219
C(Fragment=1)	-6.39266	0.49332	-1.95863
C(Fragment=1)	-6.12726	1.87575	-1.96418
O(Fragment=1)	-8.01634	-1.36333	-1.99212
C(Fragment=1)	-7.04931	-2.07107	-2.76881
N(Fragment=1)	-0.70201	2.6152	-0.2268
O(Fragment=1)	0.09121	2.24427	-1.28062
O(Fragment=1)	-0.86202	1.52863	0.59341
C(Fragment=1)	-5.59433	6.17493	-2.56603
N(Fragment=1)	-5.48096	7.25055	-2.94036
H(Fragment=1)	-3.16005	2.26053	0.89747
H(Fragment=1)	-1.20741	3.76955	-2.59102
H(Fragment=1)	-3.30912	4.83921	-3.31117
H(Fragment=1)	-5.3301	3.11964	0.04324
H(Fragment=1)	-7.82429	4.95178	-2.14103
H(Fragment=1)	-9.33952	2.98562	-2.03564

H(Fragment=1)	-9.78573	0.61186	-2.00139
H(Fragment=1)	-5.58454	-0.20747	-1.95066
H(Fragment=1)	-5.12654	2.23119	-1.95047
H(Fragment=1)	-7.27933	-3.11507	-2.75878
H(Fragment=1)	-7.07286	-1.71207	-3.77689
H(Fragment=1)	-6.07585	-1.91522	-2.35891
C(Fragment=2)	3.80177	-1.27299	-0.15156
C(Fragment=2)	4.8818	-2.01535	-0.55633
C(Fragment=2)	6.2027	-1.67651	-0.24661
C(Fragment=2)	6.4315	-0.69378	0.70576
C(Fragment=2)	5.34386	0.0864	1.11574
C(Fragment=2)	4.03758	-0.1759	0.64339
I(Fragment=2)	2.42311	1.12594	0.99253
C(Fragment=2)	7.01357	-2.59036	-1.19547
C(Fragment=2)	8.25538	-2.93373	-1.6438
C(Fragment=2)	9.71183	-3.40721	-1.37683
C(Fragment=2)	10.33985	-4.22114	-0.46279
C(Fragment=2)	10.41594	-2.89012	-2.4531
C(Fragment=2)	11.45244	-4.9086	-0.95121
H(Fragment=2)	9.98568	-4.34845	0.53436
C(Fragment=2)	11.51918	-3.51942	-2.93473
H(Fragment=2)	10.06049	-1.97599	-2.93365
C(Fragment=2)	11.96645	-4.61389	-2.25208
H(Fragment=2)	11.9095	-5.67928	-0.38287
H(Fragment=2)	11.98588	-3.17872	-3.84044
O(Fragment=2)	12.95868	-5.44201	-2.87316
C(Fragment=2)	14.23006	-4.97903	-2.48865
H(Fragment=2)	14.31843	-5.06351	-1.42861
H(Fragment=2)	14.9875	-5.56136	-2.96254
H(Fragment=2)	14.33937	-3.95245	-2.77583
H(Fragment=2)	6.30267	-3.16599	-1.74952
H(Fragment=2)	8.19552	-2.81419	-2.66155

F(Fragment=2)	7.64597	-0.50435	1.26157
F(Fragment=2)	4.68567	-3.12395	-1.29369
F(Fragment=2)	5.53278	1.17116	1.88955
F(Fragment=2)	2.57787	-1.60138	-0.59213

Coordinates for the optimised geometry of NO_2-C_1···F_4Az

Charge = 0; multiplicity = 1 in supermolecule

Charge = 0; multiplicity = 1 in fragment 1.

Charge = 0; multiplicity = 1 in fragment 2.

C(Fragment=1)	-0.55763	1.44208	-0.00476
C(Fragment=1)	0.624	1.67957	-0.73307
C(Fragment=1)	0.5616	2.33266	-1.97001
C(Fragment=1)	-0.64007	2.89913	-2.38863
C(Fragment=1)	-1.80365	2.71825	-1.64228
C(Fragment=1)	-1.79277	1.93869	-0.47141
C(Fragment=1)	-3.09442	3.47612	-2.09842
C(Fragment=1)	-4.33247	2.92659	-2.07016
C(Fragment=1)	-4.56478	1.40594	-1.99776
C(Fragment=1)	-5.87865	0.91615	-1.99982
C(Fragment=1)	-6.13377	-0.41654	-1.98217
C(Fragment=1)	-5.08413	-1.3394	-1.9839
C(Fragment=1)	-3.7536	-0.88566	-1.95034
C(Fragment=1)	-3.4882	0.49677	-1.95589
O(Fragment=1)	-5.37728	-2.74231	-1.98383
C(Fragment=1)	-4.41025	-3.45006	-2.76052
N(Fragment=1)	1.93705	1.23622	-0.21851
O(Fragment=1)	2.73027	0.86528	-1.27233
O(Fragment=1)	1.77704	0.14965	0.6017
C(Fragment=1)	-2.95527	4.79595	-2.55774
N(Fragment=1)	-2.8419	5.87157	-2.93207
H(Fragment=1)	-0.52099	0.88155	0.90576

H(Fragment=1)	1.43165	2.39057	-2.58273
H(Fragment=1)	-0.67006	3.46023	-3.30288
H(Fragment=1)	-2.69104	1.74066	0.05153
H(Fragment=1)	-5.18523	3.5728	-2.13274
H(Fragment=1)	-6.70046	1.60664	-2.02735
H(Fragment=1)	-7.14667	-0.76712	-1.9931
H(Fragment=1)	-2.94548	-1.58646	-1.94237
H(Fragment=1)	-2.48748	0.85221	-1.94218
H(Fragment=1)	-4.64027	-4.49406	-2.75049
H(Fragment=1)	-4.4338	-3.09105	-3.7686
H(Fragment=1)	-3.43679	-3.2942	-2.35062
C(Fragment=2)	6.43959	-2.65586	-0.13375
C(Fragment=2)	7.51962	-3.39822	-0.53852
C(Fragment=2)	8.84052	-3.05938	-0.2288
C(Fragment=2)	9.06932	-2.07665	0.72357
C(Fragment=2)	7.98168	-1.29647	1.13355
C(Fragment=2)	6.6754	-1.55877	0.66119
I(Fragment=2)	5.06093	-0.25693	1.01034
C(Fragment=2)	12.34965	-4.79008	-1.35902
C(Fragment=2)	12.97766	-5.60401	-0.44499
C(Fragment=2)	13.05376	-4.273	-2.43529
C(Fragment=2)	14.09026	-6.29148	-0.9334
H(Fragment=2)	12.62349	-5.73133	0.55217
C(Fragment=2)	14.15699	-4.90229	-2.91692
H(Fragment=2)	12.69831	-3.35886	-2.91585
C(Fragment=2)	14.60426	-5.99677	-2.23427
H(Fragment=2)	14.54732	-7.06216	-0.36507
H(Fragment=2)	14.6237	-4.56159	-3.82263
O(Fragment=2)	15.5965	-6.82488	-2.85535
C(Fragment=2)	16.86788	-6.3619	-2.47084
H(Fragment=2)	16.95625	-6.44638	-1.4108
H(Fragment=2)	17.62532	-6.94423	-2.94473

H(Fragment=2)	16.97718	-5.33532	-2.75802
F(Fragment=2)	10.28379	-1.88723	1.27938
F(Fragment=2)	7.32348	-4.50683	-1.27588
F(Fragment=2)	8.1706	-0.21171	1.90736
F(Fragment=2)	5.21568	-2.98425	-0.57433
N(Fragment=2)	9.65139	-3.97324	-1.17766
N(Fragment=2)	10.8932	-4.3166	-1.626

Coordinates for the optimised geometry of NO_2-C_1···F_3Az

Charge = 0 multiplicity = 1 in supermolecule

Charge = 0 multiplicity = 1 inFragment 1.

Charge = 0 multiplicity = 1 inFragment 2.

C(Fragment=1)	-0.47762	1.29386	0.05353
C(Fragment=1)	0.72865	1.58812	-0.60634
C(Fragment=1)	0.71321	2.36876	-1.7666
C(Fragment=1)	-0.48265	2.93974	-2.20776
C(Fragment=1)	-1.6793	2.68248	-1.53428
C(Fragment=1)	-1.6931	1.80718	-0.43603
C(Fragment=1)	-2.9679	3.39913	-2.01294
C(Fragment=1)	-4.20606	2.86072	-1.93157
C(Fragment=1)	-4.44405	1.33822	-1.83403
C(Fragment=1)	-5.7624	0.85619	-1.80578
C(Fragment=1)	-6.01166	-0.47797	-1.81027
C(Fragment=1)	-4.95852	-1.39989	-1.81054
C(Fragment=1)	-3.63019	-0.9437	-1.79676
C(Fragment=1)	-3.37183	0.43632	-1.78831
O(Fragment=1)	-5.2504	-2.80279	-1.84075
C(Fragment=1)	-4.26817	-3.50156	-2.61144
N(Fragment=1)	2.00737	1.08412	-0.08205
O(Fragment=1)	2.84908	0.82784	-1.12056

O(Fragment=1)	1.79803	-0.05694	0.62444
C(Fragment=1)	-2.81747	4.68054	-2.55719
N(Fragment=1)	-2.69442	5.72909	-3.00254
H(Fragment=1)	-0.46633	0.68321	0.93219
H(Fragment=1)	1.61914	2.53372	-2.31355
H(Fragment=1)	-0.48505	3.57222	-3.07142
H(Fragment=1)	-2.61554	1.54716	0.04036
H(Fragment=1)	-5.05941	3.51275	-1.97943
H(Fragment=1)	-6.57816	1.54711	-1.80416
H(Fragment=1)	-7.02366	-0.8278	-1.80332
H(Fragment=1)	-2.82214	-1.64421	-1.78118
H(Fragment=1)	-2.36343	0.79539	-1.76904
H(Fragment=1)	-4.49525	-4.54789	-2.61292
H(Fragment=1)	-4.2768	-3.13435	-3.61704
H(Fragment=1)	-3.30081	-3.34708	-2.1836
C(Fragment=2)	6.31469	-2.71096	-0.18594
C(Fragment=2)	7.36024	-3.46518	-0.7745
C(Fragment=2)	8.68048	-2.99534	-0.67015
C(Fragment=2)	8.94284	-1.93932	0.22155
C(Fragment=2)	7.89787	-1.27981	0.85826
C(Fragment=2)	6.58225	-1.59835	0.56662
I(Fragment=2)	5.02142	-0.37204	1.25321
C(Fragment=2)	11.96547	-4.89113	-1.67692
C(Fragment=2)	12.62212	-5.72401	-0.80925
C(Fragment=2)	12.70901	-4.31639	-2.71691
C(Fragment=2)	13.90558	-6.21466	-1.12412
H(Fragment=2)	12.18255	-6.0033	0.11621
C(Fragment=2)	13.96383	-4.83149	-3.04631
H(Fragment=2)	12.32902	-3.50498	-3.29893
C(Fragment=2)	14.47805	-5.88109	-2.3501
H(Fragment=2)	14.44239	-6.83859	-0.42802
H(Fragment=2)	14.52674	-4.42003	-3.86057

O(Fragment=2)	15.57985	-6.64841	-2.87158
C(Fragment=2)	16.87187	-6.19661	-2.51409
H(Fragment=2)	16.98814	-6.24333	-1.45394
H(Fragment=2)	17.60308	-6.82664	-2.98201
H(Fragment=2)	16.99988	-5.19198	-2.84735
F(Fragment=2)	7.03781	-4.60557	-1.42189
F(Fragment=2)	8.16966	-0.31306	1.78002
F(Fragment=2)	5.02029	-3.06843	-0.358
H(Fragment=2)	9.93189	-1.66463	0.44991
N(Fragment=2)	9.96477	-3.47125	-1.44251
N(Fragment=2)	10.43156	-4.64976	-1.39947

Coordinates for the optimised geometry of NO_2-C_1···F_2Az

Charge = 0; multiplicity = 1 in supermolecule

Charge = 0; multiplicity = 1 in fragment 1.

Charge = 0; multiplicity = 1 in fragment 2.

C(Fragment=1)	-5.43684	-0.38283	-0.56551
C(Fragment=1)	-4.44057	-0.54611	0.41059
C(Fragment=1)	-4.71437	-1.33481	1.53081
C(Fragment=1)	-5.98925	-1.90028	1.72092
C(Fragment=1)	-7.02785	-1.59797	0.82963
C(Fragment=1)	-6.74235	-0.85612	-0.33328
C(Fragment=1)	-8.45553	-2.15326	1.06168
C(Fragment=1)	-9.52104	-1.4605	0.61471
C(Fragment=1)	-9.49163	0.08601	0.39172
C(Fragment=1)	-10.44638	0.633	-0.46382
C(Fragment=1)	-10.45142	2.00056	-0.77157
C(Fragment=1)	-9.601	2.8957	-0.11527
C(Fragment=1)	-8.64134	2.38895	0.78521

C(Fragment=1)	-8.56856	0.97556	1.02835
O(Fragment=1)	-9.7245	4.29113	-0.43483
C(Fragment=1)	-8.64697	5.10323	0.02677
N(Fragment=1)	-3.12655	0.03255	0.22356
O(Fragment=1)	-2.21571	-0.69932	0.87507
O(Fragment=1)	-2.81477	0.04311	-1.08089
C(Fragment=1)	-8.61613	-3.54433	1.7508
N(Fragment=1)	-8.75091	-4.71177	2.32913
H(Fragment=1)	-5.21847	0.11367	-1.48023
H(Fragment=1)	-3.96197	-1.50916	2.26349
H(Fragment=1)	-6.17554	-2.57651	2.56333
H(Fragment=1)	-7.52111	-0.68392	-1.06164
H(Fragment=1)	-10.43765	-1.98509	0.41503
H(Fragment=1)	-11.18792	-0.00178	-0.8874
H(Fragment=1)	-11.13257	2.36952	-1.51553
H(Fragment=1)	-7.97508	3.05746	1.28927
H(Fragment=1)	-7.82971	0.59788	1.71275
H(Fragment=1)	-8.74995	6.09967	-0.35003
H(Fragment=1)	-8.66258	5.12873	1.08606
H(Fragment=1)	-7.72235	4.70966	-0.30013
C(Fragment=2)	3.35835	0.44447	-0.17558
C(Fragment=2)	4.6878	0.43683	0.25178
C(Fragment=2)	5.55128	-0.5693	-0.13462
C(Fragment=2)	5.13495	-1.51313	-1.12197
C(Fragment=2)	3.76395	-1.54026	-1.50463
C(Fragment=2)	2.88379	-0.56643	-1.01988
I(Fragment=2)	0.85809	-0.60028	-1.54861
C(Fragment=2)	9.13417	0.5791	0.76236
C(Fragment=2)	9.80842	1.80448	0.49722
C(Fragment=2)	9.85812	-0.58071	0.93904
C(Fragment=2)	11.20275	1.86218	0.74889
H(Fragment=2)	9.28003	2.67907	0.14937

C(Fragment=2)	11.23375	-0.53295	1.11519
H(Fragment=2)	9.33831	-1.50948	0.94727
C(Fragment=2)	11.89651	0.69796	1.11841
H(Fragment=2)	11.73107	2.78579	0.66492
H(Fragment=2)	11.77507	-1.43775	1.25222
O(Fragment=2)	13.26862	0.76214	1.50345
C(Fragment=2)	14.11241	0.63992	0.38679
H(Fragment=2)	13.90284	1.42521	-0.29234
H(Fragment=2)	15.13715	0.69882	0.68437
H(Fragment=2)	13.94227	-0.29523	-0.08313
F(Fragment=2)	3.28929	-2.50697	-2.32626
F(Fragment=2)	2.5401	1.4386	0.22586
H(Fragment=2)	5.8229	-2.22004	-1.56911
N(Fragment=2)	6.84582	-0.63144	0.55519
N(Fragment=2)	7.66436	0.45968	0.93155
H(Fragment=2)	5.0503	1.20267	0.88315

Coordinates for the optimised geometry of complex NO_2-C_1···F_2'Az

Charge = 0; multiplicity = 1 in supermolecule

Charge = 0; multiplicity = 1 in fragment 1.

Charge = 0; multiplicity = 1 in fragment 2.

C(Fragment=1)	-5.46415	-0.32047	-0.52736
C(Fragment=1)	-4.45539	-0.55024	0.41093
C(Fragment=1)	-4.71367	-1.3403	1.55958
C(Fragment=1)	-5.99021	-1.90431	1.73667
C(Fragment=1)	-7.01268	-1.58027	0.86999
C(Fragment=1)	-6.76428	-0.80746	-0.29232
C(Fragment=1)	-8.43977	-2.11293	1.08363
C(Fragment=1)	-9.50835	-1.44809	0.58495
C(Fragment=1)	-9.49705	0.08421	0.36378

C(Fragment=1)	-10.4559	0.6218	-0.48378
C(Fragment=1)	-10.48031	1.99665	-0.76552
C(Fragment=1)	-9.5972	2.87248	-0.13307
C(Fragment=1)	-8.64597	2.36163	0.76425
C(Fragment=1)	-8.55513	0.95984	0.98668
O(Fragment=1)	-9.76287	4.29733	-0.38887
C(Fragment=1)	-8.62681	5.07815	0.03967
N(Fragment=1)	-3.11342	0.02395	0.2268
O(Fragment=1)	-2.21186	-0.75399	0.84232
O(Fragment=1)	-2.81953	0.03821	-1.08582
C(Fragment=1)	-8.62017	-3.49448	1.79749
N(Fragment=1)	-8.77195	-4.65678	2.39805
H(Fragment=1)	-5.25486	0.19486	-1.44391
H(Fragment=1)	-3.9518	-1.5376	2.26833
H(Fragment=1)	-6.16437	-2.59577	2.52037
H(Fragment=1)	-7.54173	-0.63269	-1.02008
H(Fragment=1)	-10.37186	-2.02994	0.31435
H(Fragment=1)	-11.18911	-0.01056	-0.9403
H(Fragment=1)	-11.18389	2.38747	-1.46766
H(Fragment=1)	-7.98206	3.04373	1.28349
H(Fragment=1)	-7.7868	0.58523	1.62604
H(Fragment=1)	-8.74123	6.07211	-0.33161
H(Fragment=1)	-8.60742	5.07453	1.09487
H(Fragment=1)	-7.71676	4.66633	-0.34325
C(Fragment=2)	3.35559	0.46284	-0.1801
C(Fragment=2)	4.66273	0.43351	0.27319
C(Fragment=2)	5.53413	-0.57737	-0.10166
C(Fragment=2)	5.13703	-1.49952	-1.10447
C(Fragment=2)	3.79521	-1.51382	-1.53434
C(Fragment=2)	2.91145	-0.54444	-1.03294
I(Fragment=2)	0.89406	-0.58874	-1.56111
C(Fragment=2)	9.14203	0.56205	0.77881

C(Fragment=2)	9.79793	1.78239	0.50158
C(Fragment=2)	9.86933	-0.58855	0.98908
C(Fragment=2)	11.1933	1.84884	0.69801
H(Fragment=2)	9.2533	2.65277	0.17759
C(Fragment=2)	11.24315	-0.53113	1.11831
H(Fragment=2)	9.35834	-1.52483	1.06034
C(Fragment=2)	11.89199	0.6983	1.08819
H(Fragment=2)	11.71905	2.77732	0.58073
H(Fragment=2)	11.79326	-1.42299	1.26843
O(Fragment=2)	13.26854	0.77744	1.48639
C(Fragment=2)	14.11133	0.63955	0.36185
H(Fragment=2)	13.89613	1.42924	-0.30825
H(Fragment=2)	15.12863	0.69452	0.68179
H(Fragment=2)	13.92558	-0.30208	-0.09045
F(Fragment=2)	5.10258	1.39209	1.09066
N(Fragment=2)	6.83453	-0.63795	0.61689
N(Fragment=2)	7.68744	0.45486	0.94056
F(Fragment=2)	6.0121	-2.37448	-1.63338
H(Fragment=2)	2.68417	1.25274	0.13539
H(Fragment=2)	3.44038	-2.25864	-2.24131

References:

S1. Gupta, R. K.; Pathak, S. K.; De, J.; Pal, S. K.; Achalkumar, A. S.; *J. Mater. Chem. C*, **2018**, *6*, 1844-1852

S2. Saccone, M.; Spengler, M.; Pfletscher, M.; Kuntze, K.; Virkki, M.; Wölper, C.; Gehrke, R.; Jansen, G.; Metrangolo, P.; Priimagi, A.; Giese, M.; *Chem. Mater.*, **2019**, *31*, 462−470.

S3. Bruce, D. W.; Metrangolo, P.; Meyer, F.; Pilati, T.; Präsang, C.; Resnati, G.; Terraneo, G.; Wainwright, S. G.; Whitwood, A. C.; *Chem. - Eur. J.*, **2010**, *16 (31)*, 9511−9524

S4. L. A. Burns, M. S. Marshall, C. D. Sherrill; *J. Chem. Theory Comput.*, 2014, **10**, 49-57.

S5. Book Chapter: ``Counterpoise Correction and Basis Set Superposition Error´´ by C. David Sherrill, School of Chemistry and Biochemistry, Georgia Institute of Technology. (http://vergil.chemistry.gatech.edu/notes/cp.pdf)
